# Fecal microbiota from patients with Parkinson's disease intensifies inflammation and neurodegeneration in A53T mice

**DOI:** 10.1111/cns.70003

**Published:** 2024-08-19

**Authors:** Huijia Yang, Yaping Shao, Yiying Hu, Jin Qian, Panpan Wang, Lulu Tian, Yang Ni, Song Li, Murad Al‐Nusaif, Cong Liu, Weidong Le

**Affiliations:** ^1^ Key Laboratory of Liaoning Province for Research on the Pathogenic Mechanisms of Neurological Diseases, The First Affiliated Hospital Dalian Medical University Dalian China; ^2^ Department of Neurology, The First Affiliated Hospital Dalian Medical University Dalian China; ^3^ Interdisciplinary Research Center on Biology and Chemistry, Shanghai Institute of Organic Chemistry Chinese Academy of Sciences Shanghai China; ^4^ Shanghai University of Medicine and Health Sciences Affiliated Zhoupu Hospital Shanghai China

**Keywords:** dopaminergic neurodegeneration, fecal microbiota transplantation, inflammation, metabolomics, Parkinson's disease

## Abstract

**Aims:**

We evaluated the potential of Parkinson's disease (PD) fecal microbiota transplantation to initiate or exacerbate PD pathologies and investigated the underlying mechanisms.

**Methods:**

We transplanted the fecal microbiota from PD patients into mice by oral gavage and assessed the motor and intestinal functions, as well as the inflammatory and pathological changes in the colon and brain. Furthermore, 16S rRNA gene sequencing combined with metabolomics analysis was conducted to assess the impacts of fecal delivery on the fecal microbiota and metabolism in recipient mice.

**Results:**

The fecal microbiota from PD patients increased intestinal inflammation, deteriorated intestinal barrier function, intensified microglia and astrocyte activation, abnormal deposition of α‐Synuclein, and dopaminergic neuronal loss in the brains of A53T mice. A mechanistic study revealed that the fecal microbiota of PD patients stimulated the TLR4/NF‐κB/NLRP3 pathway in both the brain and colon. Additionally, multiomics analysis found that transplantation of fecal microbiota from PD patients not only altered the composition of the gut microbiota but also influenced the fecal metabolic profile of the recipient mice.

**Conclusion:**

The fecal microbiota from PD patients intensifies inflammation and neurodegeneration in A53T mice. Our findings demonstrate that imbalance and dysfunction in the gut microbiome play significant roles in the development and advancement of PD.

## Graphical abstract

FMT from PD patients altered the structure and composition of the gut microbiota in recipient mice and significantly altered the metabolic profiles, which collectively may aggravate intestinal and systemic inflammation, cause BBB damage, and eventually lead to dopaminergic neurodegeneration. In conclusion, our results suggest that gut microbiota and intestinal ecological disorders are important driving factors for the progression of PD.

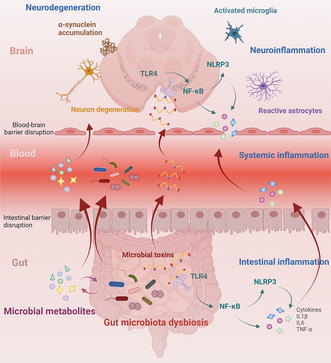

## 1 INTRODUCTION

Parkinson's disease (PD) is the second most prevalent neurodegenerative disorder.^1^ Globally, the incidence of PD is on the rise due to the aging population.^2^ PD is characterized by motor symptoms, including resting tremor, rigidity, bradykinesia, and gait/postural instability, as well as nonmotor manifestations.^3^ Gastrointestinal (GI) dysfunctions are among the most prevalent nonmotor symptoms that occur in the initial phases and even in the pre‐motor stage of PD, affecting around 80% of patients with PD.^4^, ^5^ In particular, constipation prevails in individuals with PD and may manifest years or even decades prior to the onset of motor symptoms associated with PD.^6^, ^7^

Accumulation of α‐Synuclein (α‐syn) and progressive degeneration of dopaminergic neurons in the substantia nigra (SN) are the main pathological hallmarks of PD.^8^ Studies by Braak et al.^9^, ^10^ have delineated the early involvement of the enteric nervous system (ENS), showcasing α‐syn pathology within the GI tract during the incipient stages of PD. Our previous study found that stereotaxic injection of colonic tissue from PD patients into the striatum of rats resulted in dopaminergic neurodegeneration and α‐syn deposition.^11^ The ENS and central nervous system (CNS) have bidirectional communication, and the gut microbiota plays a crucial role in facilitating the connection between the gut and the brain. Furthermore, recent reports showed that patients with neurodegenerative diseases have altered gut microbiota.^12^, ^13^ The gut microbiota is increasingly recognized to play a critical role in several neurodegenerative diseases.^12^ Multiple pathogenetic mechanisms by which the gut microbiota communicates with the CNS have been suggested. Alterations in the gut microbiota can affect the immune system, vagus nerve, ENS, neuroendocrine system, and circulatory system, all of which may have a profound impact on neurodegeneration.^14^, ^15^

Multiple studies have demonstrated that patients with PD exhibit alterations in their gut microbiota.^16^, ^17^ One or more alterations in particular bacteria may be connected with PD symptoms or pathology. There was a correlation between the abundance of *Enterobacteriaceae* and the severity of postural instability and gait impairment.^18^ Probiotics are live bacteria that, when consumed in sufficient quantities, help restore the balance of the gut microbiota, improve the integrity of the intestinal barrier, and modulate the immune system for the benefit of the host.^19^ The use of probiotics was effective in alleviating constipation symptoms in individuals with PD.^20^ Given that GI dysfunction, such as constipation and dysbiosis of the gut microbiota, may play a role in the pathological progression of PD, the utilization of probiotics may alleviate complications.^21^ Fecal microbiota transplantation (FMT) is a medical procedure transferring the fecal matter from healthy donors into the GI tract of patients.^21^ The purpose of FMT is to treat certain diseases by restoring a balanced and stable population of microorganisms in the gut.^22^ Several clinical trials have shown that FMT can effectively alleviate symptoms in PD patients.^23^, ^24^

Furthermore, the gut microbiota processes a variety of substances, yielding diverse metabolites, including short‐chain fatty acids (SCFAs), hormones, neurotransmitters, and others. These metabolites exert profound influences on neural transmission, inflammatory response, neuroprotection, and cellular apoptosis,^25^ displaying a strong correlation with the progression of PD. Our present study aimed to ascertain whether gut microbiomes sourced from PD patients can modulate disease outcomes upon the transfer into α‐syn A53T transgenic (TG) mice of PD and their age‐matched wild‐type (WT) littermates. In addition, we performed 16S ribosomal RNA (rRNA) gene sequencing and untargeted metabolomic analysis using liquid chromatography‐mass spectrometry (LC–MS) to excavate the mechanisms underlying the effects of the microbiota.

## 2 METHODS

### 2.1 Study design

In order to investigate the potential impacts of PD gut microbiota transplantation on the gut and brain function of recipients, we orally fed fecal microbiota from healthy controls (HCs, *n* = 4) or patients with PD (*n* = 4) into A53T and WT mice by gavage (100 μL per mouse, once daily, 4 weeks). The experimental design was schematically illustrated in Figure S1. First of all, we examined the motor functions of A53T and WT mice at three ages: 3‐, 9‐, and 18‐month‐old. We found that A53T mice did not develop motor dysfunction at 9 months (Figure S2), consistent with the previous study.^26^ Because our study aimed to investigate the potential impact of gut microbiota on disease development, we chose the presymptomatic mice at 7 months of age (*n* = 8–10) to start the experiments. The A53T and WT mice were randomized into four groups: WT mice treated with feces from HC (WT_HC) or from PD (WT_PD), and TG mice treated with feces from HC (TG_HC) or from PD (TG_PD). After 4 weeks of treatment, the impacts of FMT from PD patients on behavior, intestinal motility, barrier function, inflammation, and the microbiota and metabolomic profiles of the mice were assessed and analyzed.

The methods are detailed in File S1.

## 3 RESULTS

### 3.1 FMT from PD patients induces motor impairments, emotional disturbance, and intestinal dysfunctions in recipient mice

**FIGURE 1:**
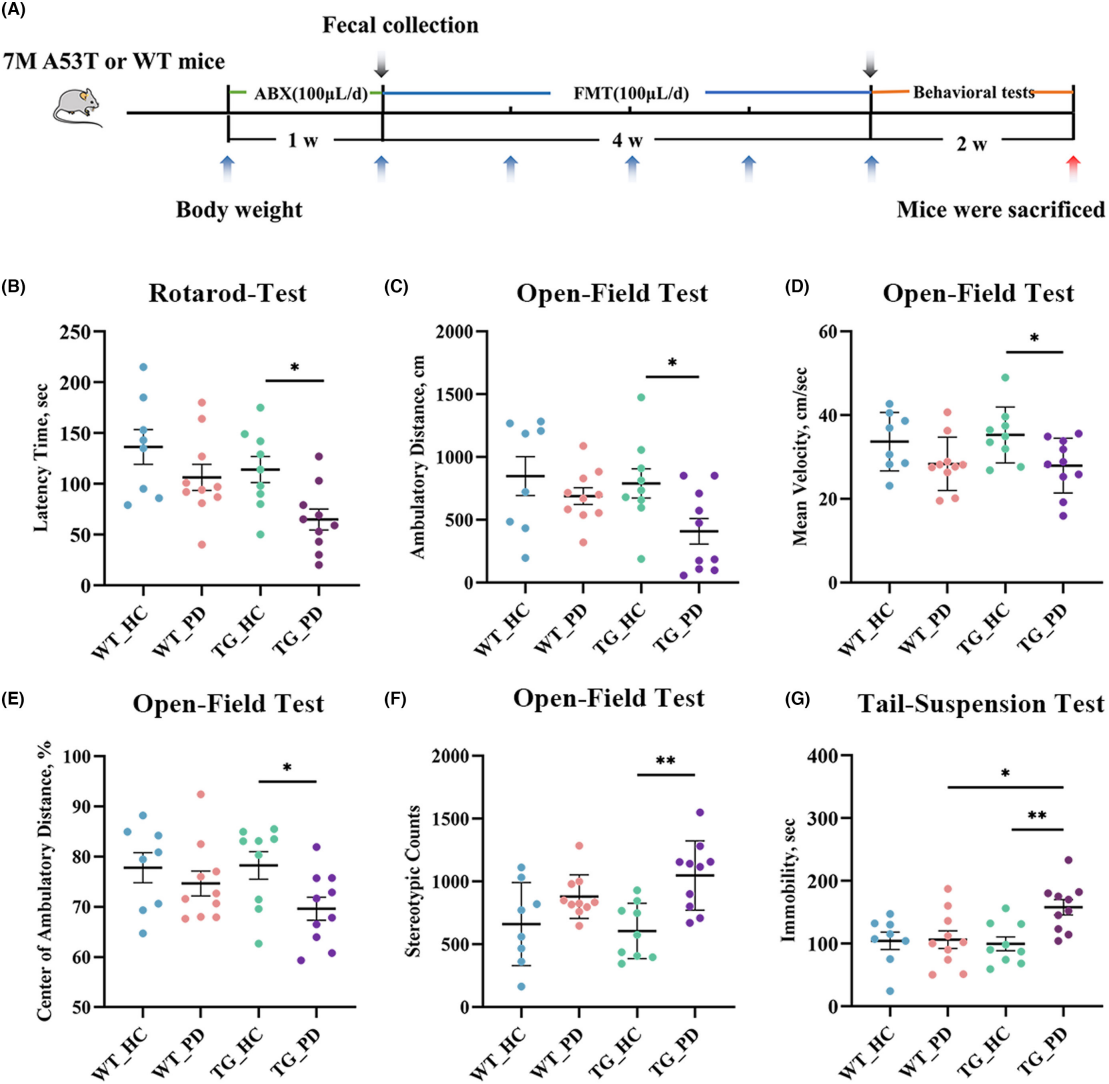
FMT from PD patients induces motor impairment and emotional alteration in recipient mice. (A) Microbiota transfer timeline overview, (B–G) Behavioral tests including Rotarod‐Test (B), Open‐Field Test (C–F) and Tail‐Suspension Test (G). *n* = 8–10 per group. *p* < 0.05 was set as the threshold for significance by two‐way ANOVA followed by post hoc comparisons using Sidak's test for multiple groups' comparisons, *p < 0.05, **p < 0.01.

Behavioral assessments were performed as shown in the timeline (Figure 1A). Motor dysfunction, as the pathological consequence of dopaminergic neuronal loss, is a major symptom in PD patients. In our present study, the rotarod‐test was used to determine whether FMT from PD patients affects the motor function of recipient mice. We found that the latency to fall off the rod was significantly decreased in the TG_PD mice compared with the TG_HC mice (Figure 1B). Consistently, in the open‐field test (OFT), ambulatory distance and mean velocity were significantly decreased in the TG_PD group compared with the TG_HC group (Figure 1C,D). The results align with previous research,^27^ demonstrating compromised motor performance in TG mice that received FMT from patients with PD.

Besides motor deficits, PD patients usually show psychiatric disorders, including anxiety and depression. In the present study, the OFT and tail‐suspension test (TST) were used to assess the mental status of mice. Compared with the TG_HC mice, the TG_PD mice showed a decrease in central distance traveled and a significant increase in stereotype counts (Figure 1E,F). Additionally, we observed that the TG_PD mice displayed severer immobility than the TG_HC mice during the TST (Figure 1G). These findings indicated that FMT from PD patients may cause or aggravate motor and nonmotor impairments.

In addition, the GI dysfunctions of the recipient mice were also assessed. Their fecal pellets were collected to measure water content, which indirectly represents colonic motility. Mice that received FMT from PD patients exhibited significantly reduced fecal wet weight and fecal water content compared with control mice (Figure S3A,B). No significant differences were observed in 24‐h food intake among all groups, excluding interference of food intake on fecal traits (Figure S3C). In addition, we measured the whole gut transit time (WGTT), which is the amount of time it takes for an edible dye to pass through the GI tract and be eliminated, to examine GI function. Mice that received the FMT from PD patients had a longer WGTT than mice that received the FMT from HCs (Figure S3D). Throughout the study, no significant change was observed in the body weight of the mice (Figure S3E).

### 3.2 FMT from PD patients induces inflammation, exacerbates pathological features, and compromises the intestinal barrier in the colon of recipient mice

**FIGURE 2:**
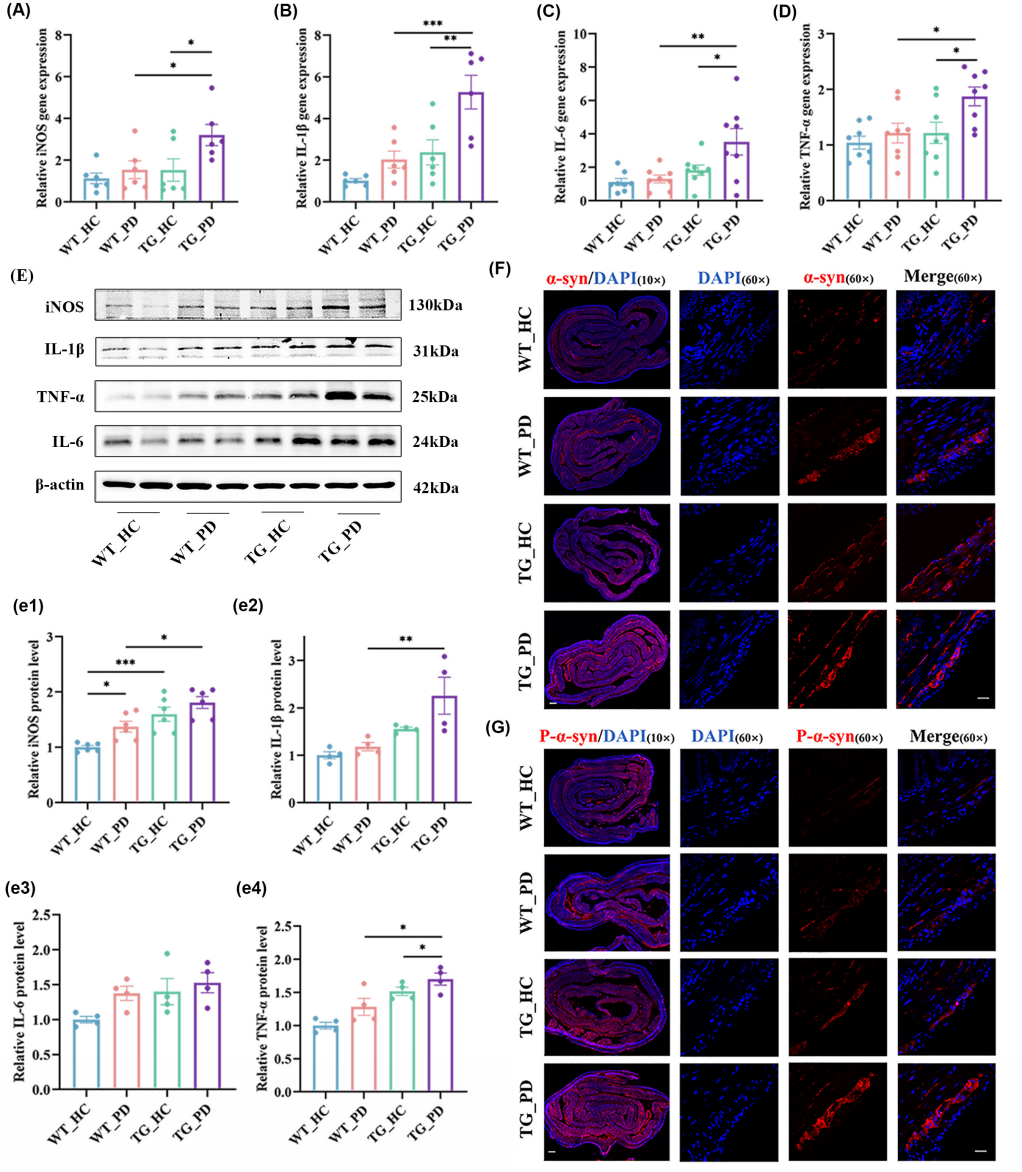
FMT from PD patients induces inflammation and aggravates pathological features in the colon in recipient mice. (A–D) The mRNA expression of iNOS (A), IL‐1β (B), IL‐6 (C), and TNF‐α (D) in the colon, *n* = 6–8 per group. (E) Representative western blot brands of iNOS, IL‐1β, TNF‐α, and IL‐6 in the colon. (e) The relative quantitative results of iNOS (e1), IL‐1β (e2), IL‐6 (e3), and TNF‐α (e4) in the colon, *n* = 4–6 per group. (F, G) Representative confocal images of α‐syn (F) and P‐α‐syn (S129, G) immunofluorescence in the colon tissue, the images in the leftmost column were acquired by 10× object lens, scale bar: 400 μm; the images in the right 3 columns were acquired transversely by 60× object lens, scale bar: 25 μm. *p* < 0.05 was set as the threshold for significance by two‐way ANOVA followed by post hoc comparisons using Sidak's test for multiple groups' comparisons, *p < 0.05, **p < 0.01, ***p < 0.001.

To further investigate the molecular interactions between gut microbial dysbiosis and gut inflammation in PD, we determined colon levels of tumor necrosis factor‐α (TNF‐α), interleukin‐1β (IL‐1β), interleukin‐6 (IL‐6), and inducible nitric oxide synthase (iNOS) by quantitative real‐time polymerase chain reaction (qRT‐PCR) and western blot. The result of qRT‐PCR showed higher levels of iNOS, IL‐1β, IL‐6, and TNF‐α in the colon of the TG mice that received FMT from PD patients compared with those received HCs (Figure 2A–D). Consistent results were obtained in western blot (Figure 2E,e), indicating intestinal inflammation through increased enteric glial reactivity in the myenteric plexuses.^28^ Our results also showed that markers of enteric glial cells, including glial fibrillary acidic protein (GFAP) and S100β, were significantly increased (Figure S4). To assess whether intestinal inflammation could aggravate PD pathologies, we performed immunofluorescence staining and found that the levels of α‐syn and phosphorylated‐α‐syn (p‐α‐syn, S129) were significantly elevated in the colon of mice that received FMT from PD compared with those received HCs (Figure 2F,G). No significant difference was observed regarding the expression level of α‐syn in dopaminergic and cholinergic neurons (Figure S5).

**FIGURE 3:**
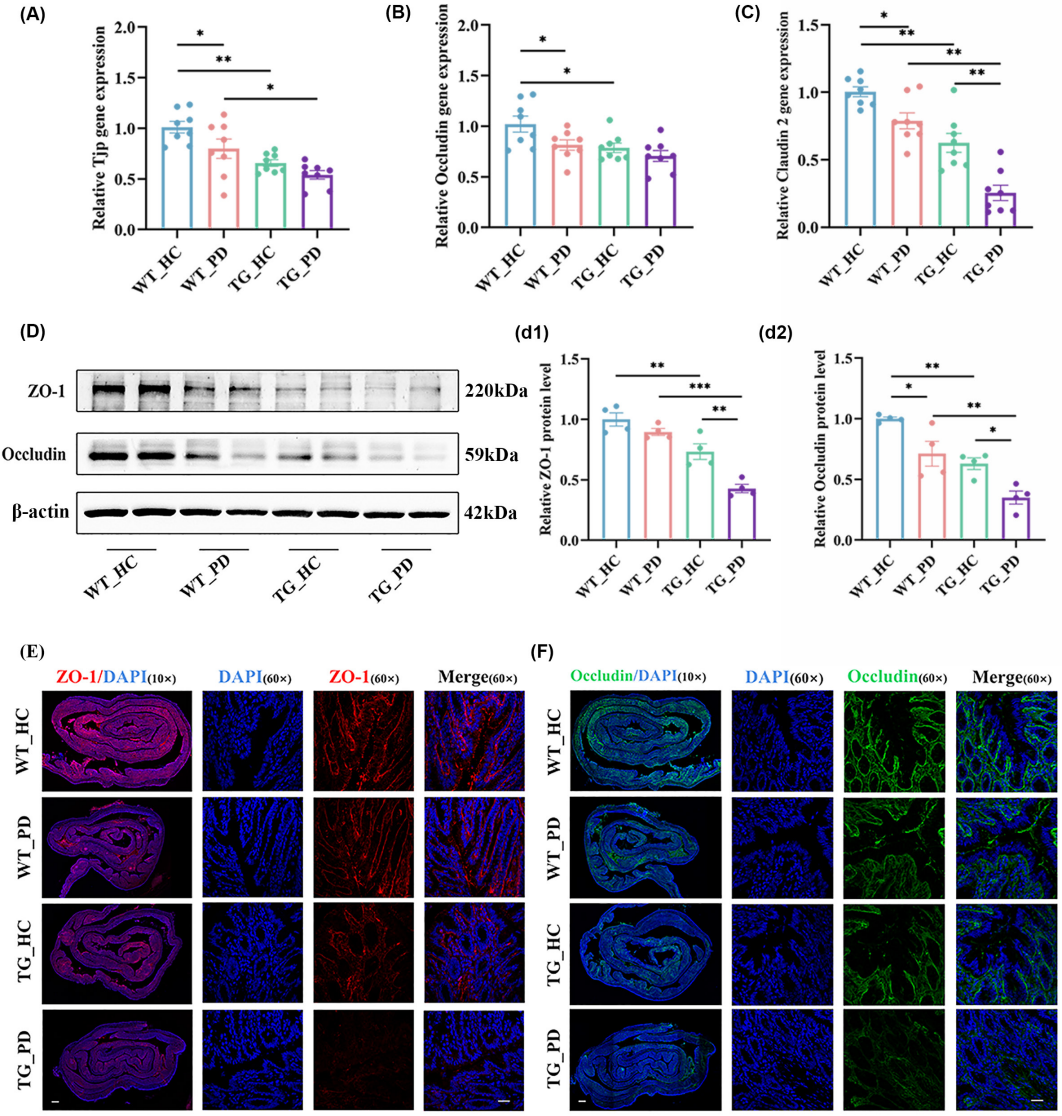
FMT from PD patients causes intestinal barrier damage in recipient mice. (A–C) The mRNA expression of Tjp (A), Occludin (B), Claudin2 (C) in the colon, *n* = 8 per group. (D) Representative western blot brands of ZO‐1 and Occludin in the colon. (d) Relative quantitative results of ZO‐1 (d1) and Occludin (d2) in the colon, *n* = 4 per group. (E, F) Representative confocal images of ZO‐1 (E) and Occludin (F) immunofluorescence of colon tissue; the images in the leftmost column were acquired by 10× object lens, scale bar: 400 μm; the images in the right 3 columns were acquired transversely by 60× object lens, scale bar: 25 μm. Data are presented as mean ± SEM. *p* < 0.05 was set as the threshold for significance by two‐way ANOVA followed by post hoc comparisons using Sidak's test for multiple groups' comparisons, *p < 0.05, **p < 0.01, ***p < 0.001.

The gut microbiota plays a crucial role in regulating and maintaining the integrity of the intestinal barrier.^29^ Alterations in the composition of the intestinal microbiota may compromise the integrity of the gut barrier, thereby contributing to the development of various diseases. Key tight junction proteins, such as Zonula occludens‐1 (ZO‐1) and Occludin, are pivotal in maintaining gut permeability. In our study, the levels of these proteins and mRNAs were determined by western blot, qRT‐PCR, and immunofluorescence staining to evaluate the effect of the FMT from PD patients on the intestinal barrier function in recipient mice. QRT‐PCR showed the tight junctions were disrupted in the colon (Figure 3A–C). Meanwhile, western blot analysis of the colon confirmed the decreased levels of tight junction proteins in the mice that received the FMT from PD patients (Figure 3D,d). Consistently, fluorescence intensities of ZO‐1 and Occludin were decreased in the colon of mice that received the FMT from PD patients (Figure 3E,F), indicating disrupted tight junction integrity.

### 3.3 FMT from PD patients increases circulating inflammation cytokines and disrupts the blood–brain barrier in recipient mice

The gut microbiota, along with activated immune cells, generates an array of inflammatory mediators that can breach the compromised intestinal barrier and stimulate the production of inflammatory cytokines by other immune cells within the systemic circulation.^30^ Peripherally derived inflammatory factors can exert a direct impact on the brain by increasing the permeability of the blood–brain barrier (BBB).^31^ Therefore, it is necessary to examine systemic inflammatory factors to understand the impact of gut microbiota derived from PD patients on inflammatory alterations in the mouse brain. In this study, we obtained peripheral blood samples from mice via orbital bleeding and determined the levels of cytokines by enzyme‐linked immunosorbent assay (ELISA). Our data revealed a substantial elevation of proinflammatory cytokines, including IL‐1β, IL‐6, and TNF‐α, in the plasma of mice administered fecal samples sourced from individuals with PD (Figure S6). To investigate the effects of feces from PD on the integrity of the BBB, we analyzed the tight junction proteins, Occludin and ZO‐1. Western blot analysis of the midbrain found that levels of tight junction proteins were decreased in mice after receiving the FMT from PD patients (Figure S7A). We also assessed the BBB marker CD‐31, an important adhesion protein of intercellular junctions in endothelial cells^32^ by immunofluorescence staining. The results showed that CD‐31 expression was down‐regulated in the cortex of mice after receiving the FMT from PD patients, indicating that the BBB was disturbed (Figure S7B).

### 3.4 FMT from PD patients induces neuroinflammation and aggravates pathological features in the SN of recipient mice

**FIGURE 4:**
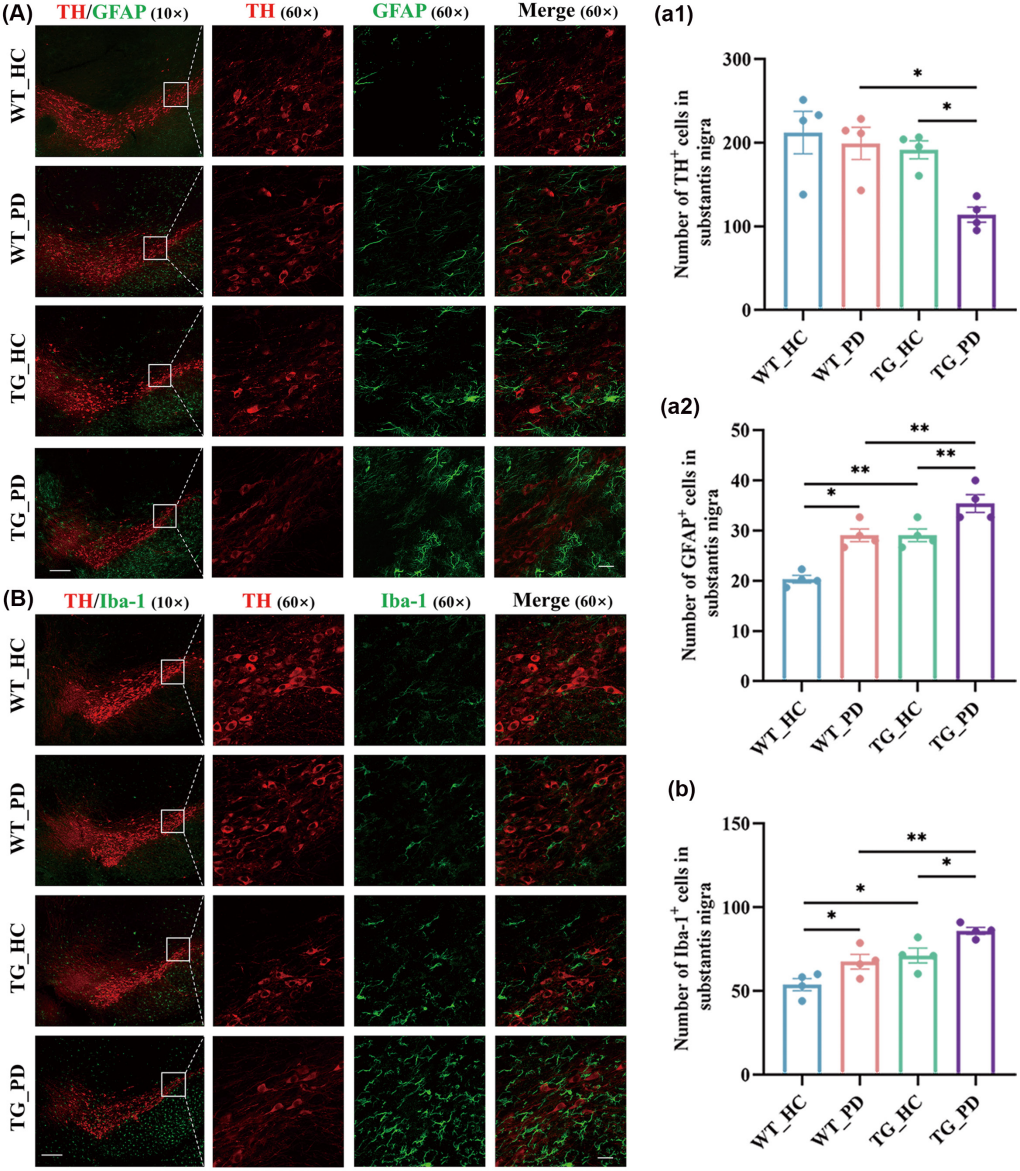
FMT from PD patients induces neuroinflammation in the SN of recipient mice. (A, B) Representative confocal images of TH (A, B), GFAP (A) and Iba‐1 (B) immunofluorescence in the SN; the images in the leftmost column were acquired by 10× object lens, scale bar: 200 μm; the images in the right 3 columns were acquired by 60× object lens, scale bar: 25 μm. (a, b) Numbers of TH cells (a1), GFAP cells (a2) and Iba‐1 cells (b) in the SN, *n* = 4 per group. Data are presented as mean ± SEM. *p* < 0.05 was set as the threshold for significance by two‐way ANOVA followed by post hoc comparisons using Sidak's test for multiple groups' comparisons, *p < 0.05, **p < 0.01.

**FIGURE 5:**
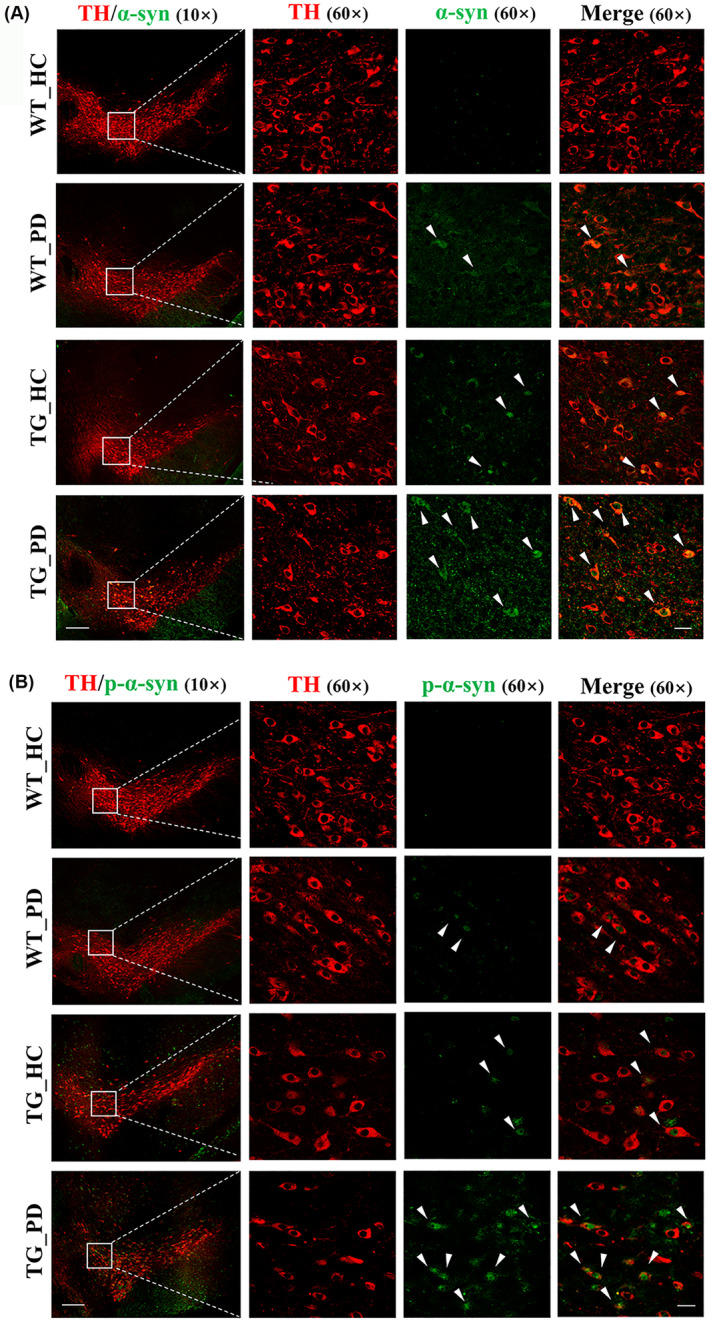
FMT from PD patients aggravates pathological features in the SN of recipient mice. (A) Representative capture of α‐syn immunofluorescence in the SN. (B) Representative captures of p‐α‐syn (S129) immunofluorescence in the SN; the images in the leftmost column were acquired by 10× object lens, scale bar: 200 μm; the images in the right 3 columns were acquired by 60× object lens, scale bar: 25 μm.

To further explore how PD fecal samples aggravated motor deficits, we detected histological characteristics and neuroinflammation in the SN. Immunofluorescence staining signified a reduced number of tyrosine hydroxylase (TH)‐positive neurons and an elevated number of GFAP and Iba‐1‐positive cells in the SN of the mice that received FMT from PD patients (Figure 4). Subsequently, we found that alterations in the gut microbiota, induced by FMT from individuals with PD, aggravated the pathological accumulation of α‐syn and p‐α‐syn (S129) within the SN and the colocalization of α‐syn or p‐α‐syn in TH‐positive neurons (Figure 5). These results suggest the possibility that exposure to the PD‐derived gut microbiota may increase neuroinflammation and exacerbate α‐syn pathology within the brain.

### 3.5 FMT from PD patients activates the TLR4/NF‐κB/NLRP3 signaling pathway in both the colon and SN of recipient mice

**FIGURE 6:**
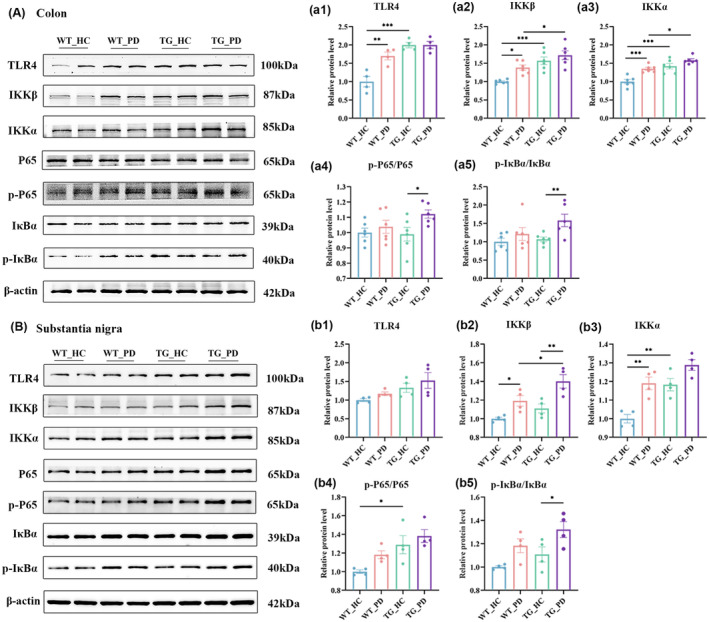
FMT from PD patients activates the NF‐κB pathway in both the colon and SN in recipient mice. (A) The representative western blot brands of TLR4, IKKβ, IKKα, P65, p‐P65, IκBα, and p‐IκBα in the colon. (a) Density analysis of TLR4 (a1), IKKβ (a2), IKKα (a3), p‐P65/P65 (a4), and p‐IκBα/IκBα (a5) in the colon, *n* = 4–6 per group. (B) The representative western blot brands of TLR4, IKKβ, IKKα, P65, p‐P65, IκBα, and p‐IκBα in the SN. (b) Density analysis of TLR4 (b1), IKKβ (b2), IKKα (b3), p‐P65/P65 (b4), and p‐IκBα/IκBα (b5) in the SN, *n* = 4 per group. Data are presented as mean ± SEM. *p* < 0.05 was set as the threshold for significance by two‐way ANOVA followed by post hoc comparisons using Sidak's test for multiple groups' comparisons, *p < 0.05, **p < 0.01, ***p < 0.001.

**FIGURE 7:**
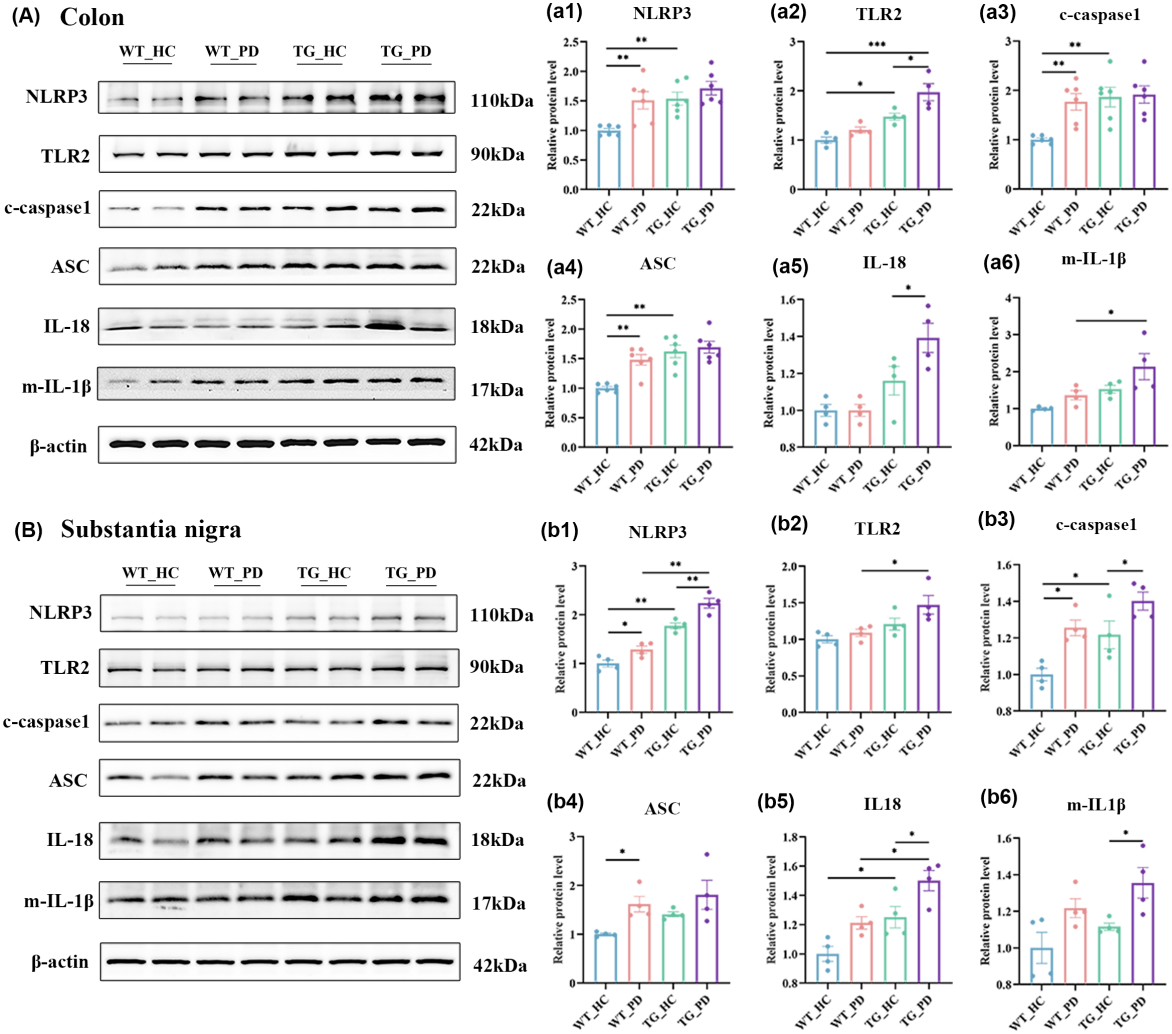
FMT from PD patients activates the NLRP3 inflammasome in both the colon and SN in recipient mice. (A) The representative western blot brands NLRP3, TLR2, c‐caspase1, ASC, IL18 and m‐IL1β in the colon. (a) Density analysis of NLRP3 (a1), TLR2 (a2), c‐caspase1 (a3), ASC (a4), IL18 (a5) and m‐IL1β (a6) in the colon, *n* = 4–6 per group. (B) The representative western blot brands of NLRP3, TLR2, c‐caspase1, ASC, IL18 and m‐IL1β in the SN. (b) Density analysis of NLRP3 (b1), TLR2 (b2), c‐caspase1 (b3), ASC (b4), IL18 (b5) and m‐IL1β (b6) in the SN, *n* = 4 per group. Data are presented as mean ± SEM. *p* < 0.05 was set as the threshold for significance by two‐way ANOVA followed by post hoc comparisons using Sidak's test for multiple groups' comparisons, *p < 0.05, **p < 0.01, ***p < 0.001.

The gut microbiota holds potential for modulating the ENS via Toll‐like receptors (TLRs) and their microbe‐associated molecular pattern ligands. Previous studies have demonstrated the significance of TLR4 signaling in regulating GI motility.^33^, ^34^ FMT from PD patients may elevate the levels of downstream inflammatory factors by activating the TLR4/NF‐ κB/NLRP3 signaling pathway. These reports suggest that the TLR4/NF‐ĸB/NLRP3 pathway plays a crucial role in mediating PD, intestinal flora, and inflammation.^35^ Indeed, we observed the activation of the TLR4/NF‐κB pathways in the colon and SN of the mice that received FMT from PD patients (Figure 6). Moreover, the mice that received FMT from PD patients showed elevated levels of caspase‐1 and mature IL‐1β, TLR2, NLRP3, and Apoptosis‐associated Speck‐like protein containing a CARD (ASC) protein in the colon and SN (Figure 7). These findings suggest that FMT from PD patients may facilitate PD pathologies and disease progression by potentiating the TLR4/NF‐κB/NLRP3 pathway.

### 3.6 FMT from PD patients alters gut microbiota composition in recipient mice

**FIGURE 8:**
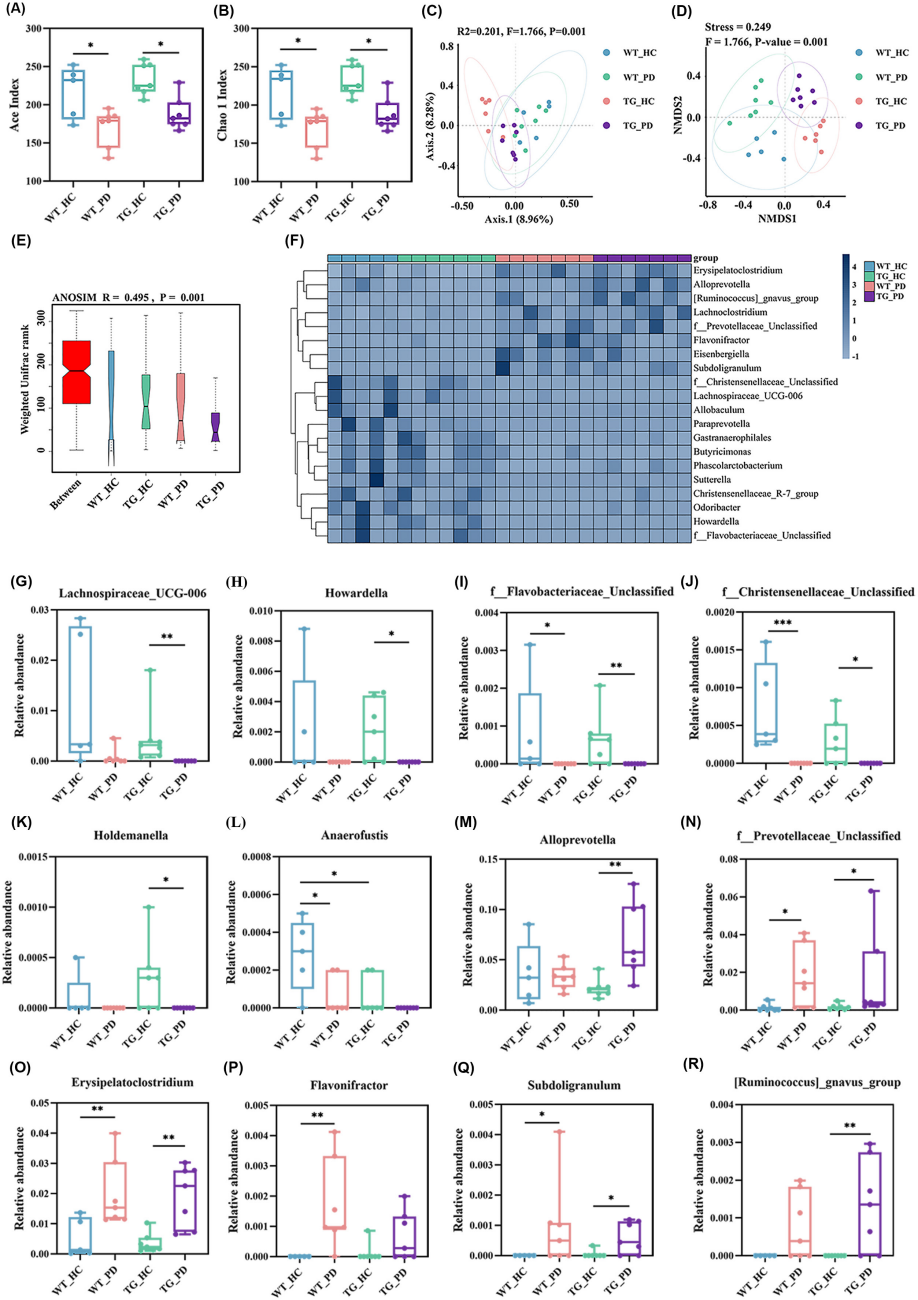
FMT from PD patients alters gut microbiota composition in recipient mice. (A, B) α‐diversity: Comparison of ace index (A) and chao1 index (B) based on ASV levels in different groups. (C, D) PCoA (C) and NMDS (D) of β‐diversity based on the ASV level, where each point represents a single sample colored by group circle. (E) Analysis of β diversity of gut microbiota by ANOSIM analysis in different groups. (F) Heatmap about relative abundances of gut microbiota at the genus level in different groups. (G–R) Bacterial genera were differentially observed in the four groups. *n* = 5–7 per group. Data were presented as medians with an interquartile range and analyzed by the Kruskal–Wallis test, *p < 0.05, **p < 0.01, ***p < 0.001.

Because our current study found that the fecal microbiota of PD patients activated the inflammatory reaction in the colon, we further investigated the composition of the gut microbiota in mice that received different treatments using 16S rRNA gene sequencing. The α‐diversity of the gut microbiota, including ace and Chao 1 index, was markedly decreased in the mice that received the FMT from PD patients compared with the mice that received HCs (Figure 8A,B). This result is consistent with the decreased α‐diversity of the gut microbiota in PD patients (Figure S8). The β‐diversity, including principal coordinate analysis (PCoA), nonmetric multidimensional scaling (NMDS), and analysis of similarity (ANOSIM), showed an obvious distinction in the gut microbiota structure between recipient mice that received fecal microbiota from different sources (Figure 8C–E). In addition, the heat map showed the differential bacteria at the genus level among mice with different treatments (Figure 8F). We further selected 12 genera and displayed their details using box plots (Figure 8G–R). A lower relative abundance of *Lachnospiraceae_UGG‐006*, *Howardella*, *f_Flavobacteriaceae_Unclassified*, *f_Christensenellaceae_Unclassified*, *Holdemanella*, and *Anaerofustis* (Figure 8G–L) as well as a higher relative abundance of *Alloprevotella*, *f_Prevotellaceae_Unclassified*, *Erysipelatoclostridium*, *Flavonifractor*, *Subdoligranulum*, *and [Ruminococcus]_gnavus* at the gene level were found in the mice that received FMT from PD patients (Figure 8M–R). Furthermore, Spearman correlation analysis was performed to identify the association between different key genera and inflammatory and barrier‐related indicators in the colon (Figure S9). Our results showed that some genera enriched in the mice that received FMT from PD patients, including *Subdoligranulum*, *Erysipelatoclostridium*, [*Ruminococcus*]*_gnavus*, *Alloprevotella*, *f_Prevotellaceae_Unclassified*, and *Flavonifractor*, were significantly correlated with inflammatory and barrier‐related indicators in the colon. The correlation coefficients for other genera that were reduced in the mice that received FMT from PD patients, such as *Holdemanella*, *Howardella*, *f_Flavobacteriaceae_Unclassified*, *Christensenellaceae_R‐7_group*, *Anaerofustis*, *Lachnospiraceae_UGG‐006*, and *f_Christensenellaceae_Unclassified*, were the opposite. These findings indicate substantial alterations in the composition and function of the gut microbiota in mice following FMT from PD patients, highlighting the pivotal role of gut microbes in PD‐related inflammatory responses.

### 3.7 FMT from PD patients alters the levels of fecal metabolites in recipient mice

**FIGURE 9:**
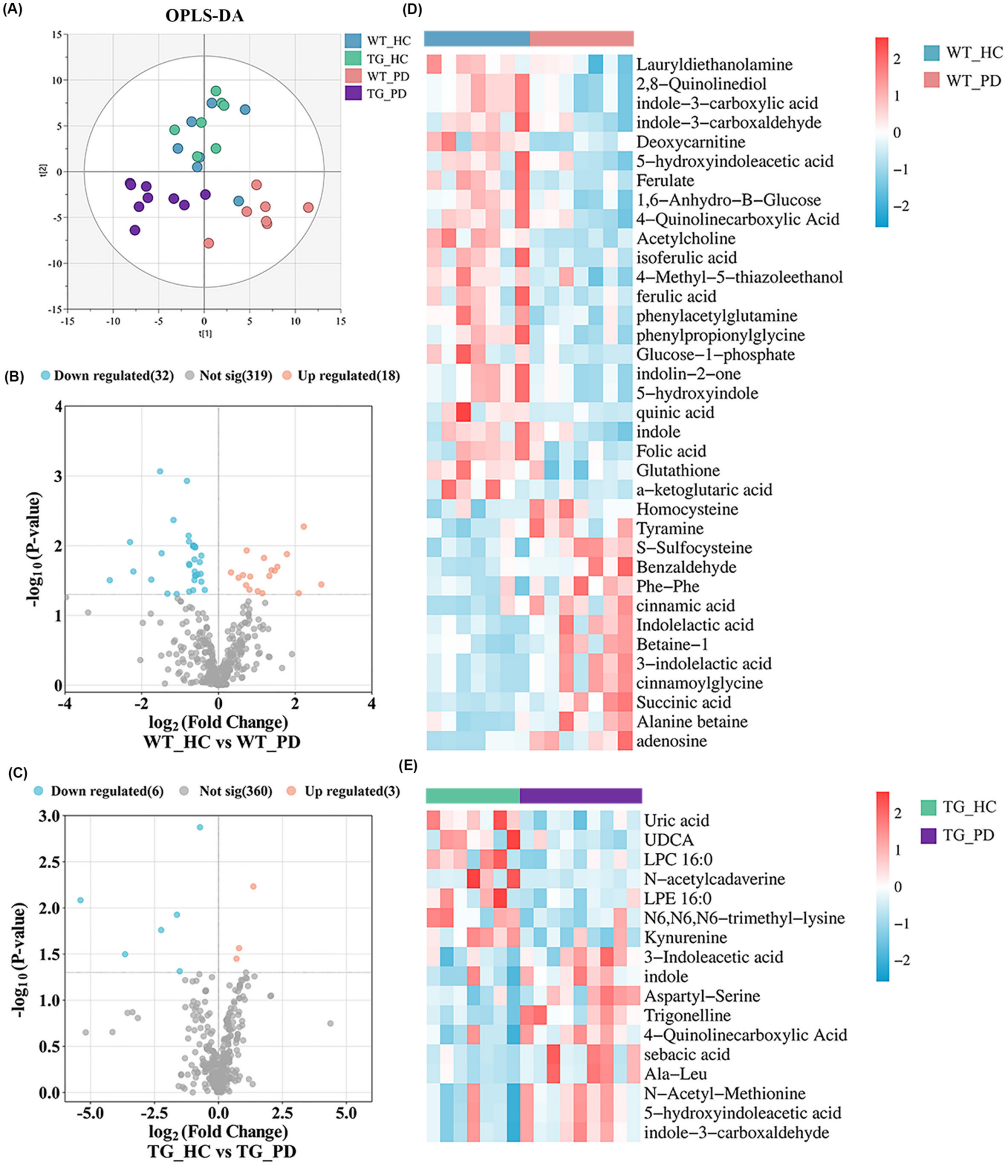
FMT from PD patients alters the fecal metabolite levels in recipient mice. (A) OPLS‐DA score plot of the metabolome in fecal of different groups of mice. (B, C) Volcano plot of the differential fecal metabolites in WT_PD (B) and TG_PD (C) filtered by univariate analysis. (D, E) Heat maps of the differential metabolites between different mice in fecal. *n* = 7–9 per group.

Given that the host's metabolic pathways are constantly regulated by the gut microbiota, the fecal metabolome is thought to be a functional readout of the gut microbiome. We used untargeted metabolomics to determine alterations in fecal metabolites. Quality control (QC) samples were analyzed through person‐correction analysis to ensure that the metabolomic data are reliable and accurate. The QC samples showed a strong correlation, indicating good reproducibility (Figure S10A). Additionally, the PLS‐DA score plots showed that the QC samples clustered closely together, providing additional evidence of trustworthy data quality (Figure S10B). OPLS‐DA showed a significant separation of samples among the different groups. Furthermore, mice that were given FMT from patients with PD exhibited a higher degree of variation in their metabolomic profile compared to mice who were given FMT from HCs (Figure 9A). The differentiated metabolites with a *p*‐value less than 0.05 were visually portrayed using distinct colors in volcano plots, based on the results of the univariate analysis (Figure 9B,C). As shown in the heat map, metabolites such as uric acid were reduced in TG mice that received FMT from PD patients, while metabolites such as ferulate were reduced in WT mice that received FMT from PD patients (Figure 9D,E). In order to investigate the relationship between fecal microbiome and fecal metabolome, the Spearman's correlation coefficient between differential fecal metabolome and differential bacterial genera was presented as a heat map (Figure S11). A total of 152 statistically significant associations between microbiota and fecal metabolites were identified, reaching a significant difference of *p* < 0.05. In conclusion, FMT from PD patients altered the structure and composition of the gut microbiota and significantly altered fecal metabolism in recipient mice.

## 4 DISCUSSION

PD is accompanied by changes in the intestinal microbial community.^36^, ^37^, ^38^ Our present findings may provide additional evidence that alterations in the gut microbiota are associated with PD. We document the decreased α‐diversity and altered gut microbiota composition in mice that received microbiota from PD patients. This result is consistent with the previous studies in PD patients^39^ and PD animal models.^40^ β‐diversity analysis revealed a distinct separation between the microbiota of mice that received FMT from PD patients and HCs. In recipient mice FMT from PD patients, we identify certain genus‐level bacteria: *Erysipelatoclostridium* ^41^ and *Ruminococcus gnavus*,^42^ which are associated with colon disease and inflammatory bowel disease. However, the abundance of other probiotics decreased in the feces of recipient mice that received FMT from PD patients. The abundance of *Holdemanella*, *Howardella*, *Lachnospiraceae_ucg‐006*, and *Anaerofustis* was positively correlated with SCFAs,^43^ which may play a role in maintaining barrier integrity and intestinal homeostasis.^44^ It has been shown that the content of *Holdemanella* was lower in the constipated PD group.^45^

In our study, we demonstrated that dysregulated gut microbiota induces intestinal inflammation, which is attributed to the NF‐κB and NLRP3 pathways. NF‐κB is a crucial nuclear transcription factor that can control the transcription level of genes associated with inflammation. External stimuli, such as lipopolysaccharide, trigger the activation of NF‐κB and cause its redistribution into the nucleus. This process stimulates the synthesis of proinflammatory cytokines.^46^ In addition, recent findings suggest that NLRP3 is also involved in maintaining intestinal homeostasis and regulating the gut microbiota.^47^ The colonic mucosa is a prominent location for NLRP3 activation and has been linked to intestinal homeostasis regulation. Its heightened expression can facilitate the caspase‐1‐mediated inflammatory cascade, subsequently triggering the expression of downstream inflammatory factors, including IL‐1β, IL‐6, and TNF‐α. NF‐κB activation not only directly promotes the expression of inflammatory cytokines such as pro‐IL‐1β, TNF‐α, and IL‐6 but also mediates the transcription of NLRP3.^48^ In this study, we observed higher levels of inflammatory factors in the colon of mice that received PD patients' microbiota compared to those of mice that received HCs, which might be induced by NF‐κB and NLRP3 inflammasome activation. In addition to the severe inflammatory reactions in the gut, we detected breakdown of the colonic epithelial barrier, including a considerable decrease in ZO‐1 and Occludin in the colon. We have further analyzed the correlation between the indicators of intestinal inflammation, the intestinal barrier, and the key bacterial genera. Their strong correlation confirmed that the dysregulated gut microbiota may aggravate intestinal inflammation and contribute to intestinal barrier damage.

Braak et al. proposed a hypothesis suggesting the potential prion‐like spread of α‐syn from the ENS to the dorsal motor nucleus of the vagus nerve and subsequently to other regions of the CNS.^9^ This hypothesis has been validated in several animal models.^49^ In addition to the vagus nerve, other pathways may exist for bidirectional communication between the gut and brain. Systemic inflammation caused by intestinal inflammation and intestinal barrier damage perturbs the BBB and is essential for gut‐brain communication.^50^ In our study, we observed elevated levels of circulating inflammatory factors in mice that received FMT from PD patients. NF‐κB and NLRP3 inflammasome are activated, which may further activate microglia and astrocytes in the brain. Overactivated microglia and astrocytes are believed to play an important role in neuroinflammation and dopaminergic neurodegeneration in the pathogenesis of PD.^51^ In addition, we found that α‐syn and p‐α‐syn, neuropathological hallmarks of PD, were abnormally deposited in the SN of the mice that received FMT from PD patients. We believe that the activation of the NLRP3 inflammasome and NF‐κB might lead to the buildup of α‐syn.^52^ Conversely, α‐syn produced by damaged dopaminergic neurons triggers the activation of NF‐κB and NLRP3 inflammasome,^53^, ^54^ establishing a positive feedback loop that exacerbates PD pathology. The relationship between gut microbiota and PD inflammation continues to be a focus of research. In PD model mice, gut microbiota dysregulation and secreted proinflammatory factors activate systemic inflammatory responses, leading to increased neuroinflammation.^55^ Our finding provides further insights into this phenomenon, demonstrating how dysregulated gut microbiota in PD patients releases proinflammatory molecules, disrupts the gut barrier, and activates circulating inflammation, ultimately exacerbating neuroinflammation. Importantly, we have observed that the levels of inflammation and α‐syn were significantly elevated in the mice after receiving FMT from PD compared with HCs. However, we have observed dopaminergic neuronal damage only in TG mice but not in WT mice. One explanation is that overexpressing α‐syn in TG mice may influence the development of PD pathology, whereas the WT mice may need more time to develop PD pathology.

The influence of the gut microbiota extends beyond the intestine as it interacts with the host to generate active metabolites transported through the circulatory system, impacting various bodily functions. Our study reveals a significant difference in fecal metabolite composition between recipient mice that received FMT from different sources. An inadequate amount of beneficial microbes and their metabolites could trigger an inflammatory reaction.^56^ In the present study, we found that succinic acid and homocysteine (Hcy) are increased, while folic acid, ferulic acid, and 5‐hydroxyindole (5‐HI) are decreased in the feces of WT mice that received FMT from PD patients. Succinic acid is a metabolic intermediate of the tricarboxylic acid (TCA) cycle that builds up in intestinal inflammation and disruption of the microbiota.^57^ Hcy is an important metabolite in methionine metabolism. Folic acid biosynthesis in the gut may also affect Hcy levels.^58^ Increased Hcy and succinic acid contributed to the development of PD and disease severity.^59^, ^60^, ^61^ Hcy can trigger the activation of nuclear factor NF‐κB in the brains of mice, leading to a rise in the release of IL‐1β, TNF‐α, and other inflammatory factors, thus intensifying the inflammatory response in the brain tissue.^62^ Ferulic acid is found in foods, beverages, supplements, and herbs, and its absorption rate depends on gut microbiota composition and activity.^63^ On the other hand, ferulic acid can regulate the composition and metabolism of the gut microbiota, significantly enhance the intestinal barrier, and reduce intestinal inflammation.^64^ 5‐HI, an intestinal microbial transformation product of dietary supplements, promotes intestinal peristalsis through its action on L‐type calcium channels.^65^ We have also observed a decreased level of ursodeoxycholic acid (UDCA) in the feces of TG mice that received FMT from PD patients. UDCA can not only inhibit the phosphorylation of NF‐κB and reduce the production of inflammatory cytokines but also maintain the integrity of the intestinal barrier by enhancing the expression of tight junction proteins.^66^ Spearman's correlation analysis in our study has shown that dominant genera are correlated with differential metabolites.

We acknowledge several limitations in our study. First, the complexity of microbiota interactions may not be fully captured in this study. Second, although we have confirmed alterations in the fecal microbiota among different groups, we have not conducted a comprehensive investigation into the specific bacteria or metabolites closely related to PD. Also, the therapeutic potential of targeting these bacteria or metabolites remains to be determined. Third, due to the incomplete replication of the complexities of the human microbiome and pathobiology in animal models, the clinical translation of animal microbiome‐based research remains challenging. The existence of these limitations emphasizes the necessity for continued research efforts to unravel the complexities of the microbiota‐gut‐brain axis and fully harness their potential.

## 5 CONCLUSION

We transplanted fecal microbiota from HCs or PD patients into a mouse model of microbiota to evaluate the critical role of gut microbiota in the pathophysiology of PD. Our work demonstrates that FMT from PD donors to recipient mice results in not only gut homeostasis alteration but also motor behavioral and pathological characteristics of PD. The additional multiomics experiments in vivo may document a profound and complex disruption of the gut‐brain axis and identify microorganisms and metabolites possibly associated with PD.

Overall, FMT from PD patients altered the structure and composition of the gut microbiota in recipient mice and significantly changed the metabolic profiles, which collectively may aggravate intestinal and systemic inflammation and eventually lead to dopaminergic neurodegeneration. Our findings suggest that imbalance, BBB damage, and dysfunction in the gut microbiome may play significant roles in the development and advancement of PD. Improving the gut microbiota with probiotics, prebiotics, and FMT may help slow down neurodegeneration by restoring the integrity of the gut barrier and reducing inflammation.

## AUTHOR CONTRIBUTIONS

HY: conducted experimental work, performed data analysis, and wrote the original draft; YS: coconducted experimental work and performed bioinformatics analysis for metabonomics; YH: helped conduct experimental work and edited and revised the manuscript; JQ: conducted clinical study and collected fecal samples from PD patients; PW, LT, and YN: helped conduct experimental work; SL, NM, and CL: edited manuscript; WL: project supervision, initiating research concept, and manuscript editing and revision. All authors agreed to be accountable for the content of the work.

## FUNDING INFORMATION

This work was supported in part by fundings from Shanghai Municipal Central Government Funds for Guiding Local Scientific and Technological Development (YDZX20213100001002), and the National Nature Science Foundation of China (32220103006 and 82271524).

## CONFLICT OF INTEREST STATEMENT

The authors declare that they have no competing interests.

## CONSENT TO PARTICIPATE

All participants provided written informed consent.

## Supporting information


File S1.

## Data Availability

The data that support the findings of this study are available from the corresponding author upon reasonable request.
