# Supplementary material for: Fecal microbiota from patients with Parkinson's disease intensifies inflammation and neurodegeneration in A53T mice
Source: CNS Neurosci Ther. 2024 Aug 19;30(8):e70003. doi: 10.1111/cns.70003 (PMC11333719; doi:10.1111/cns.70003)
Supplement: Supplementary file 1 — File S1. [file CNS-30-e70003-s001.docx]

**Methods**

**Preparation of fecal transplant microbiota**

Human subjects were enrolled at the First Affiliated Hospital of Dalian Medical University. This study received ethical approval from the Ethics Committee of the First Affiliated Hospital of Dalian Medical University and written informed consent was obtained from all participants. We collected fecal samples from four human subjects diagnosed with PD, as well as four matched HCs. Fecal samples from the donor were mechanically homogenized in equal amounts under an anaerobic condition, and the mixed feces were added to sterile saline (100 mg:1 mL) and filtered through a sterile 100μm filter to prepare fecal bacteria suspension^1^. The bacteria suspension was then collected and stored in aliquots at -80 °C until use.

**Animals and treatment**

TG mice expressing the human A53T mutant of α-synuclein driven by the prion promoter and WT mice were obtained from the Jackson Laboratory (Bar Harbor, ME, USA). The mice were housed under standard conditions (room temperature 22±1°C, relative humidity 50±10%, 12 to 12-hour light/dark cycle with light on at 8:00 AM) with food and water ad libitum. The animal experiments were carried out in compliance with the rules set by the Institutional Animal Care Committee of Dalian Medical University, China. To ensure the re-establishment of transplanted gut microbiota^2^, all mice were given a combination of antibiotics, specifically 1 g/L ampicillin, 0.5 g/L vancomycin, 1 g/L neomycin, and 1 g/L metronidazole (referred to as Abx) prior to FMT (100 μL/day, 7 days). The decreased α-diversity of feces microbiota in mice after antibiotics treatment suggest that antibiotics substantial depleted gut microbiota (**Figure S12**). FMT was performed immediately after the intestinal microbiota substantial depletion. Four FMT groups were inoculated with a bacterial suspension at a daily dose of 100 μL for a duration of 4 weeks. It is worth noting that we employed a mixture of 4 human subjects’ fecal specimens to investigate the impacts of PD microbiota. Therefore, these amalgamated samples exhibited greater diversity and did not emulate any individual fecal sample. The mice were weighed at “Pre-Abx,” “Post-Abx”, “1 week post-FMT”, “2 week post-FMT”, “3 weeks post-FMT”, and “4 weeks post-FMT” time points. The collection of fresh fecal samples was performed at 9:00 a.m. to avoid the impact of circadian rhythms on the microbiome. The fecal samples were thereafter transferred to a sterile microtube, which was promptly submerged in liquid nitrogen and preserved at -80 °C until required. After FMT, mouse behavior and GI transit were assessed. Mice were sacrificed thereafter, and tissues were collected for analysis.

**Rotarod-test**

As described previously^3^, mice were tested on the Rotarod apparatus (IITC Life Science, Woodland Hills, CA) to assess their motor coordination and balance abilities. Animals were tested on the rotating rod with the speed automatically accelerating from 4 to 40 r/min over a period of 5 minutes. The latency to fall from the rotating rod was recorded. All animals were pre-trained at 10 r/min for 5 consecutive days prior to the test, which was conducted three times a day at intervals of 1 hour.

**Open-field test**

The open-field test (OFT) was performed to investigate the spontaneous motor activity and exploratory behavior of rodents. Mice were placed in a locomotor activity monitor, which consisted of a 25×25 cm square arena surrounded by 30 cm walls and equipped with a computer-controlled photocell (Med Associates Inc., St. Albans, USA). Over 5-minute trials, total distance traveled, mean velocity, stereotypic counts, and distance traveled in the center were recorded as indices of spontaneous activity and depression or anxiety-like behavior, respectively. Following each test, sanitize the arena using a 75% ethanol solution to eradicate any scent clues.

**Tail-suspension test**

The tail-suspension test (TST) was employed to evaluate depressive-like behavior. The tails of mice were affixed to adhesive tape, positioned 2 cm from the tip of the tail, with the mouse head 30 cm above the plane of the laboratory bench. The duration of immobility was measured for a period of 6 minutes.

**Intestinal transit assessment**

**Whole Gut Transit Time**

The mice were housed separately in their homecage without access to food or water for an hour. Whole Gut Transit Time (WGTT) was measured after oral gavage of a 0.1 mL 4% (w/v) brilliant blue food dye solution in 0.5% methylcellulose. Following the delivery of brilliant blue food dye, the time spent by each mouse to produce a blue fecal pellet was recorded and analyzed.

**Fecal pellets output**

Each mouse was housed individually in homecage for 1 hour. Pellets produced during this period were gathered, and the overall wet weight was evaluated. Subsequently, the pellets were subjected to an overnight drying process at a temperature of 60°C, after which their weight in the dry state. The fecal water content was determined using the following formula: fecal water content (%) = (fecal wet weight - dry weight) / fecal wet weight × 100.

**Food intake test**

To assess food intake, we removed each animal from its original cage and housed them individually for 24 hours, providing free access to food and water. We quantified food intake by measuring the difference between the quantities weighed before and after the test.

**Quantitative real-time polymerase chain reaction (qRT-PCR)**

Total RNA was extracted from homogenized colon tissues with TRIzol Reagent (Invitrogen, Waltham, MA) and reverse-transcribed into cDNA using the cDNA Synthesis Kit (Vazyme #R333, Nanjing, China). QPCR was performed using the SYBR Green qPCR Master Mix Kit (#A021A, TaKaRa Biotechnology, Dalian, China). β-actin was used as the reference gene. The relative mRNA level was normalized to β-actin. The primer sequences used are listed in **Table S1**.

**Western blot**

The mixture of RIPA lysate, protease inhibitors, and phosphatase inhibitors was used to lyse the SN and colon tissues. Protein concentration was measured by the BCA method. Equal samples were separated by electrophoresis in 10% or 12% sodium dodecyl sulfate-polyacrylamide gels and transferred to 0.45-μm polyvinylidene difluoride membranes (IBFP0812C; Millipore, Burlington, MA). After blocking with 5% nonfat milk, the membranes were incubated overnight at 4 °C with the primary antibodies, followed by 1 hour of incubation at room temperature with the horseradish peroxidase-conjugated secondary antibody. The blots were then visualized with the super-sensitive electrochemiluminescence luminescence reagent (MeilunBio, Dalian, China). The data was quantitatively evaluated using ImageJ software (National Institutes of Health). Details of the antibodies used in this study are listed in **Table S2**.

**Immunofluorescence staining and confocal microscopy observation**

The distal colon and brain were fixed with 4% paraformaldehyde overnight at 4 °C, then incubated in 30% sucrose at 4 °C until equilibrated and embedded in an optimal cutting temperature compound. Next, coronal sections (40 μm and 10 μm thickness for brain and colon, respectively) were cut on a freezing microtome (Leica, Wetzlar, Germany). Tissue slides were incubated with blocking buffer (10% normal goat serum, 1% bovine serum albumin, 0.3% Triton X-100, PBS solution) for 1 hour at room temperature and then incubated with the primary antibodies overnight at 4 °C. Following the incubation with the primary antibody, the slides were washed with PBS and incubated with secondary antibodies. The fluorescence was observed and captured by confocal microscopy (A1 confocal, Nikon Instruments (Japan) Co., Ltd.). The fluorescent density was evaluated using ImageJ software. The antibodies used here we are listed in **Table S2**.

**Enzyme-linked immunosorbent assay (ELISA)**

Peripheral blood samples were collected into ethylene diamine tetra-acetic acid-containing microtubes and subsequently subjected to centrifugation, leading to the immediate separation of the plasma (the uppermost layer) for subsequent analysis. Following the protocols provided by the manufacturer (Elabscience, Wuhan, China), the concentrations of plasma inflammatory cytokines, including interleukin-1β (IL-1β), interleukin-6 (IL-6), and tumor necrosis factor-α (TNF-α), were determined using ELISA kits.

**16S rRNA sequencing**

Fecal pellets from recipient mice were collected, bacterial DNA was extracted, and 16S rRNA sequencing was performed. Alpha diversity, including ace and Chao 1 index, was used for the analysis of species diversity in a single sample. Principal coordinate analysis (PCoA), nonmetric multidimensional scaling (NMDS), and analysis of similarity (ANOSIM) were employed to assess beta diversity by comparing microbial compositions across samples. A comparison was made between groups to determine the relative abundance of taxa within the gut microbiota at the genus level. The data were presented as medians with an interquartile range and analyzed by the Kruskal-Wallis test.

**Metabolomics analysis**

**Biological sample pretreatment**

For fecal sample, after 24 h of lyophilization, approximately 10 mg of feces were weighed and placed into a 2 mL centrifuge tube with a pre-added zirconia grinding bead of 6-mm i.d. Then, 300 μL of ultrapure water was added and vortexing for 2 min. Homogenization was performed by adding 300 μL of methanol with spiked ISs (Table S3) using a mixed grinding apparatus (MM-400, Retsch Technology, Han, Germany) at 30 Hz for 1 min (5 times). After incubation at room temperature for 10 min and centrifugation at 18,000 g for 15 min (4°C), the resulting supernatant was collected and passed through a nylon filter (diameter of 13 mmm and pore size of 0.22 μm). A 250-μL aliquot was lyophilized and reconstituted in 70 μL 20% acetonitrile (v/v).

**Metabolomics analysis**

Metabolomics analyses was conducted using an Ultra Performance Liquid Chromatography (UPLC, Waters, Manchester, UK) coupled with tripleTOF^TM^ 5600 plus (Applied Biosystems, Foster City, CA) MS system. For feces, extracts were retained and gradient eluted from an ACQUITY UPLC BEH C8 column using ultrapure water and acetonitrile with 0.1% formic acid solution in the positive ionization mode. An ACQUITY UPLC HSS T3 column using ultrapure water and 95% methanol containing 6.5 mM ammonium bicarbonate was used for the negative ionization mode. Detailed chromatographic and MS conditions have been described in our previous study^4^. To maximize the coverage of gut microbiota-associated metabolites, a Supelco Discovery HS-F5 column (Merk, Darmstadt, Germany) was also used for feces analysis. A 5-μL sample aliquot was injected with the column temperature at 40°C and the flow rate was set at 0.25 mL min^-1^. The mobile phases of ultrapure water and acetonitrile with 0.1% formic acid were used in the positive ionization mode. The elution gradient was started with 0% B for 2 min, and linearly increased to 60 % B within 18 min, to 100% B within 3 min and held for 3 min, then went back to 0 % B in 0.1 min, followed by 4 min of equilibration. Detailed chromatographic and MS conditions for lipidomics analysis are available from our previous work ^5,6^.

**Raw data preprocessing**

Metabolites identification based on accurate mass, chromatographic retention and tandem mass spectrometry (MS/MS) fragmentation patterns were performed by comparing the qualitative information of each metabolite with that of reference chemical standards in an in-house database using both OSI/SMMS and MS-DIAL (Ver.5.1) ^7,8^. The peak area of each metabolite was manually integrated using the SCIEX OS (Ver.2.1.6.59781) software. The raw data were normalized to the total intensity of all detected ions in each sample before further statistical analysis.

**Statistical analysis**

For statistical analysis, Prism 8.0 and SPSS were utilized. All data points represent different samples. All data except 16S rRNA gene sequencing data were analyzed by a one-way or two-way ANOVA. Differences were considered statistically significant at *p* < 0.05. Shapiro-Wilk normality test was used to determine the normality of the data. The statistics regarding microbiome analysis were described in the 16S rRNA sequencing. For metabolome, the *t* test was used to determine differential metabolites (*p*<0.05). SIMCA-P 14.1 software (Umetric AB, Umea, Sweden) was used for metabolome analyses.

**References**

1. Yang Y, Zheng X, Wang Y, et al. Human Fecal Microbiota Transplantation Reduces the Susceptibility to Dextran Sulfate Sodium-Induced Germ-Free Mouse Colitis. *Front Immunol*. 2022;13:836542. doi:10.3389/fimmu.2022.836542

2. Bárcena C, Valdés-Mas R, Mayoral P, et al. Healthspan and lifespan extension by fecal microbiota transplantation into progeroid mice. *Nat Med*. Aug 2019;25(8):1234-1242. doi:10.1038/s41591-019-0504-5

3. Wang P, Chen X, Wang Y, et al. Essential role for autophagy protein VMP1 in maintaining neuronal homeostasis and preventing axonal degeneration. *Cell Death Dis*. Jan 22 2021;12(1):116. doi:10.1038/s41419-021-03412-5

4. Shao Y, Li T, Liu Z, et al. Comprehensive metabolic profiling of Parkinson's disease by liquid chromatography-mass spectrometry. *Mol Neurodegener*. Jan 23 2021;16(1):4. doi:10.1186/s13024-021-00425-8

5. Shao Y, Fu Z, Wang Y, et al. A metabolome atlas of mouse brain on the global metabolic signature dynamics following short-term fasting. *Signal Transduction and Targeted Therapy*. 2023;8(1)doi:10.1038/s41392-023-01552-y

6. Li J, Ren S, Piao HL, et al. Integration of lipidomics and transcriptomics unravels aberrant lipid metabolism and defines cholesteryl oleate as potential biomarker of prostate cancer. *Sci Rep*. Feb 11 2016;6:20984. doi:10.1038/srep20984

7. Zhao X, Zeng Z, Chen A, et al. Comprehensive Strategy to Construct In-House Database for Accurate and Batch Identification of Small Molecular Metabolites. *Analytical Chemistry*. 2018/06/19 2018;90(12):7635-7643. doi:10.1021/acs.analchem.8b01482

8. Tsugawa H, Ikeda K, Takahashi M, et al. A lipidome atlas in MS-DIAL 4. *Nat Biotechnol*. Oct 2020;38(10):1159-1163. doi:10.1038/s41587-020-0531-2

**Table S1. List of primers used for quantitative real-time PCR assays.**

| **mRNA** | **Forward （5’-3’）** | **Reverse（5’-3’）** |
| --- | --- | --- |
| β-actin | CTGTGCCCATCTACGAGGGCTAT | TTTGATGTCACGCACGATTTCC |
| IL-1β | AATGCCACCTTTTGACAGTGAT | TGCTGCGAGATTTGAAGCTG |
| IL-6 | CCCCAATTTCCAATGCTCTCC | GGATGGTCTTGGTCCTTAGCC |
| TNF-α | CGTCAGCCGATTTGCTATCT | CGGACTCCGCAAAGTCTAAG |
| iNOS | AATCTTGGAGCGAGTTGTGG | AATCTCTGCCTATCCGTCTCG |
| Tjp | TTCCCAGCTTATGAAAGGGTT | TCGCTTCTTTCAGGGCACCGTA |
| Ocln | ACGGACCCTGACCACTATGA | TCAGCAGCAGCCATGTACTC |
| Cldn2 | TGGTTCCTGACAGCATGAAA | CTTTGGGCTGTTGAGCAGAT |

**Table S2. List of primary antibodies for Western blotting (WB) and Immunofluorescent staining (IF).**

| **Antibody** | **Company** | **Application** |
| --- | --- | --- |
| β-actin | Cell Signaling Technology | WB |
| GAPDH | Cell Signaling Technology | WB |
| iNOS | Abcam | WB |
| IL-1β | Cell Signaling Technology | WB |
| TNF-α | Proteintech | WB |
| IL-6 | Santa | WB |
| α-syn | BD bioscience | IF |
| P-α-syn(S129) | Abcam | IF |
| ZO-1 | Santa | WB, IF |
| Occludin | Santa | WB, IF |
| CD-31 | Proteintech | IF |
| TH | Millipore | WB, IF |
| GFAP | Dako, Abcam | WB, IF |
| ChAT | Proteintech | IF |
| Iba-1 | Wako, Proteintech | WB, IF |
| TLR4 | Santa | WB |
| IKKβ | Cell Signaling Technology | WB |
| IKKα | Cell Signaling Technology | WB |
| NF-κB | Cell Signaling Technology | WB |
| p-NF-κB | Cell Signaling Technology | WB |
| IκBα | Cell Signaling Technology | WB |
| p-κBα | Cell Signaling Technology | WB |
| NLRP3 | Cell Signaling Technology | WB |
| TLR2 | Abcam | WB |
| c-caspase1 | Wanlei | WB |
| ASC | Cell Signaling Technology | WB |
| IL18 | Wanlei | WB |
| m-IL1β | Wanlei | WB |

**Table S3. Concentrations of the stable isotope labeled internal standards in methanol for metabolite extraction in feces.**

| **Internal standards** | **Concentration (μg/mL)** |
| --- | --- |
| Carnitine C2:0-d_3_ | 0.50 |
| Carnitine C8:0-d_3_ | 0.50 |
| Carnitine C12:0-d_3_ | 0.50 |
| Carnitine C16:0-d_3_ | 0.50 |
| LPC 19:0 | 3.00 |
| FFA C16:0-d_3_ | 7.50 |
| CDCA-d_4_ | 4.50 |
| CA-d_4_ | 6.00 |
| Tryptophan-d_5_ | 13.00 |
| Phenylalanine-d_5_ | 11.00 |
| Leucine-d_3_ | 4.50 |
| Choline-d_4_ | 6.00 |
| Alanine-d_3_ | 3.00 |
| Hippuric acid-d_5_ | 1.56 |


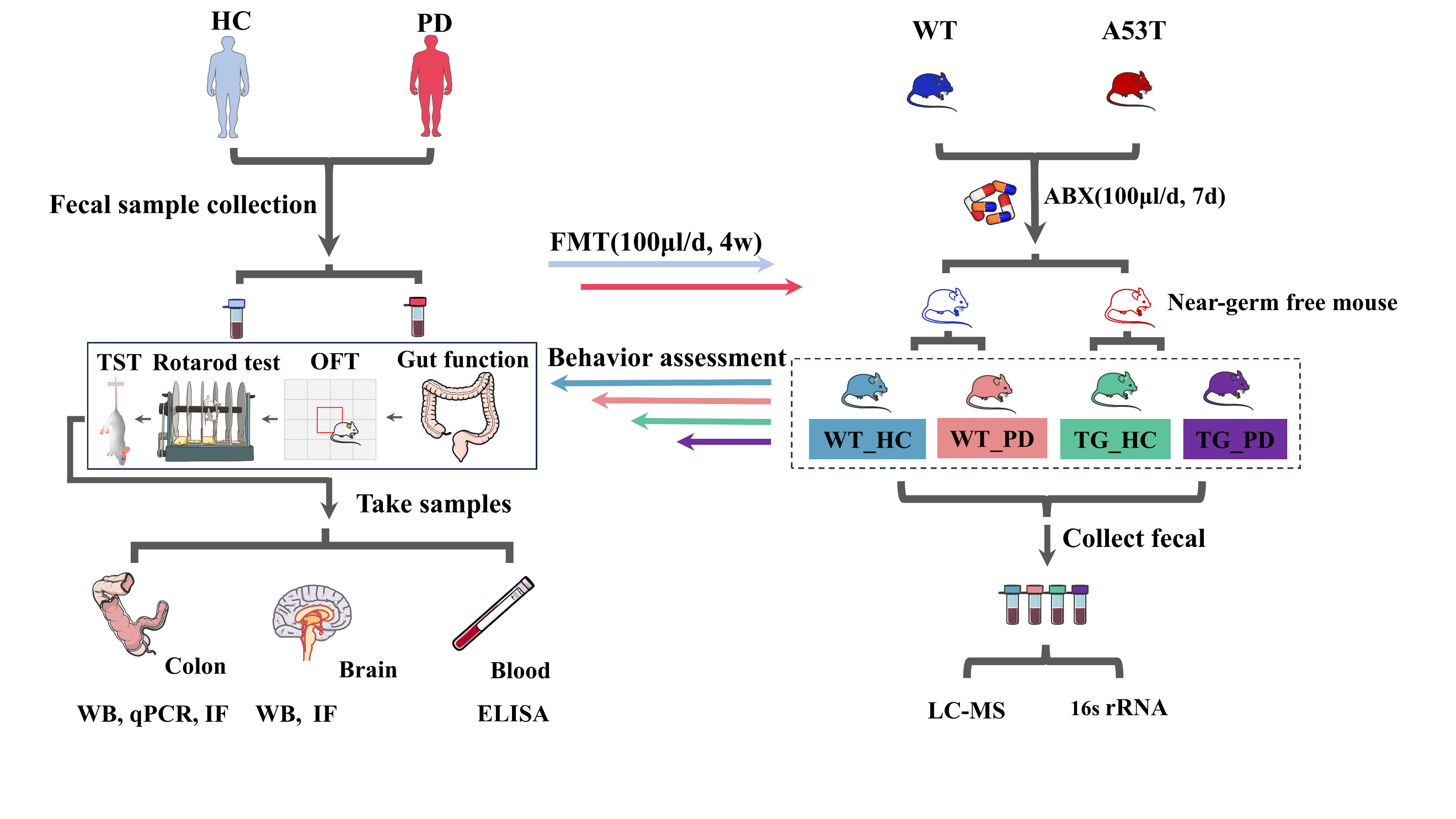


**Figure S1**

**The experimental design for FMT.** All mice were given a combination of antibiotics (referred to as Abx) prior to FMT (100 μL per mouse, once daily, 1 week). Orally fed fecal microbiota from HCs or patients with PD into A53T and WT mice by gavage (100 μL per mouse, once daily, 4 weeks). After 4 weeks of treatment, the impacts of FMT from PD patients on behavior, intestinal motility, barrier function, inflammation, and the microbiota and metabolomic profiles of the mice were assessed and analyzed.


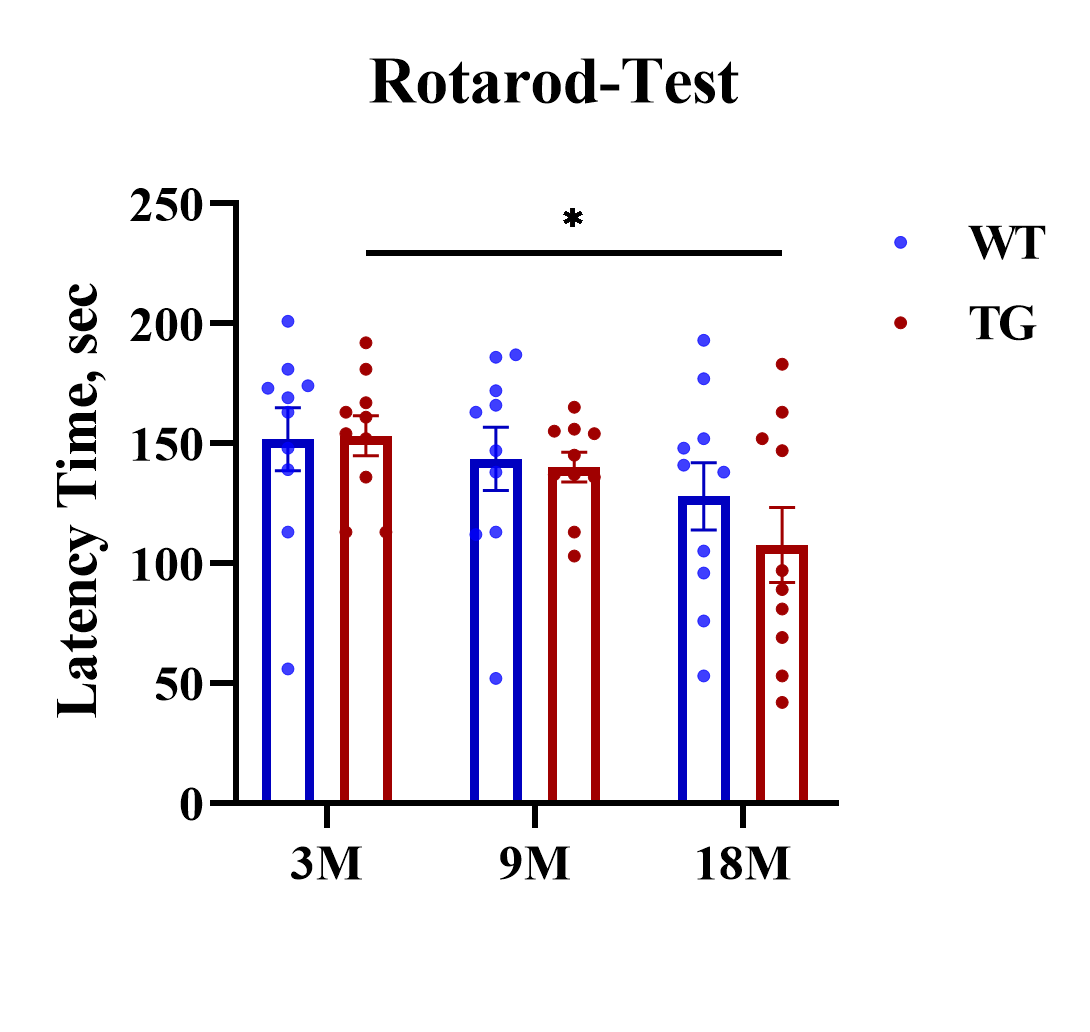


**Figure S2**

**Rotarod test in mice of different months.** Latency time of mice of different months in the rotarod test. n =10 per group. Data are presented as mean ± SEM. *p* < 0.05 was set as the threshold for significance by two-way ANOVA followed by post hoc comparisons using Sidak’s test for multiple groups’ comparisons, **p* < 0.05.


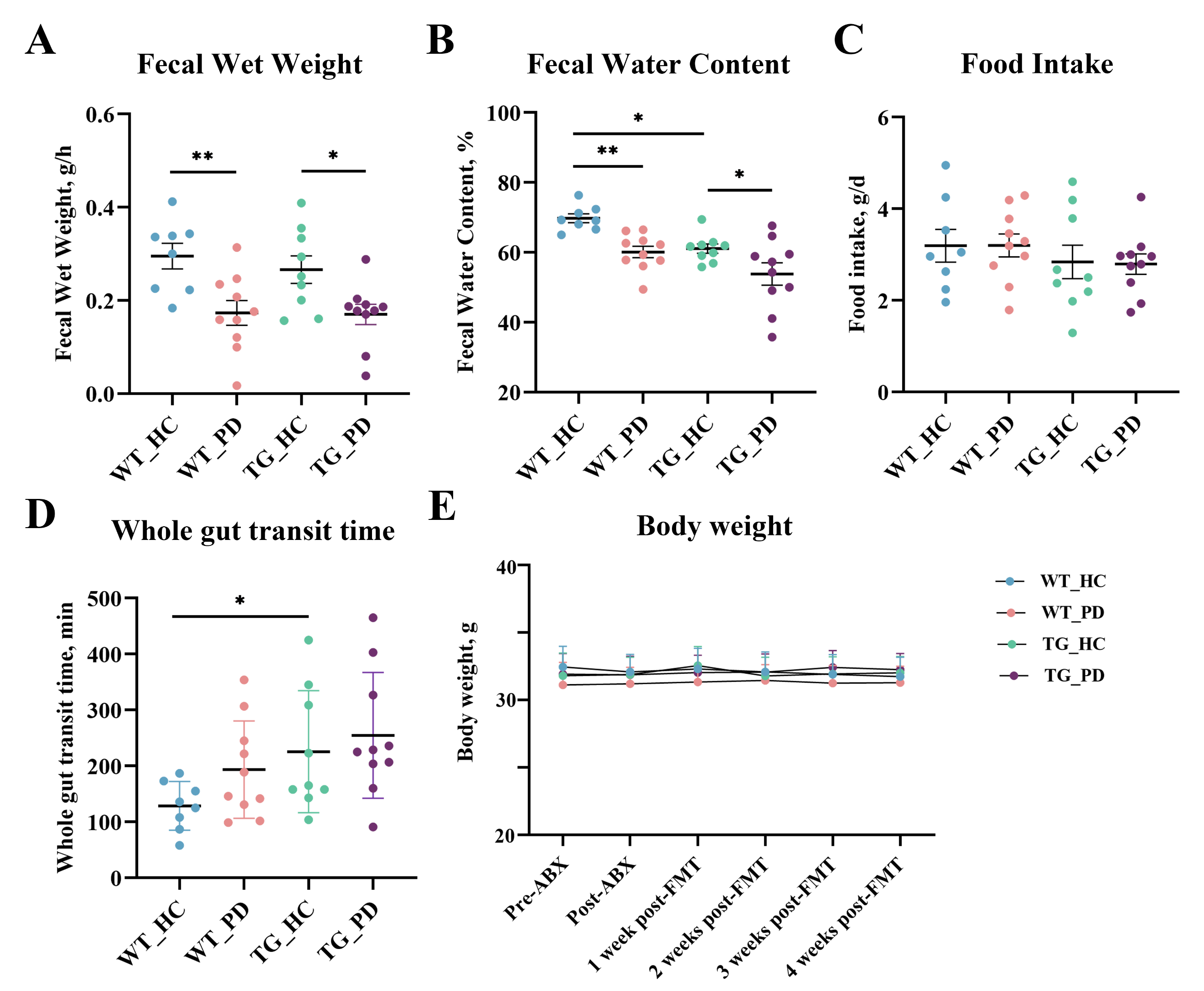


**Figure S3**

**FMT from PD patients induces intestinal dysfunction in recipient mice. A-B** Detection of fecal indices, including fecal wet weight **(A)** and fecal water content **(B)**. **C** Food intake. **D** Whole gut transit time. **E** The body weights of mice. n = 8-10 per group. Data are presented as mean ± SEM. *p* < 0.05 was set as the threshold for significance by two-way ANOVA followed by post hoc comparisons using Sidak’s test for multiple groups’ comparisons, **p* < 0.05, ***p* < 0.01, ****p* < 0.001.


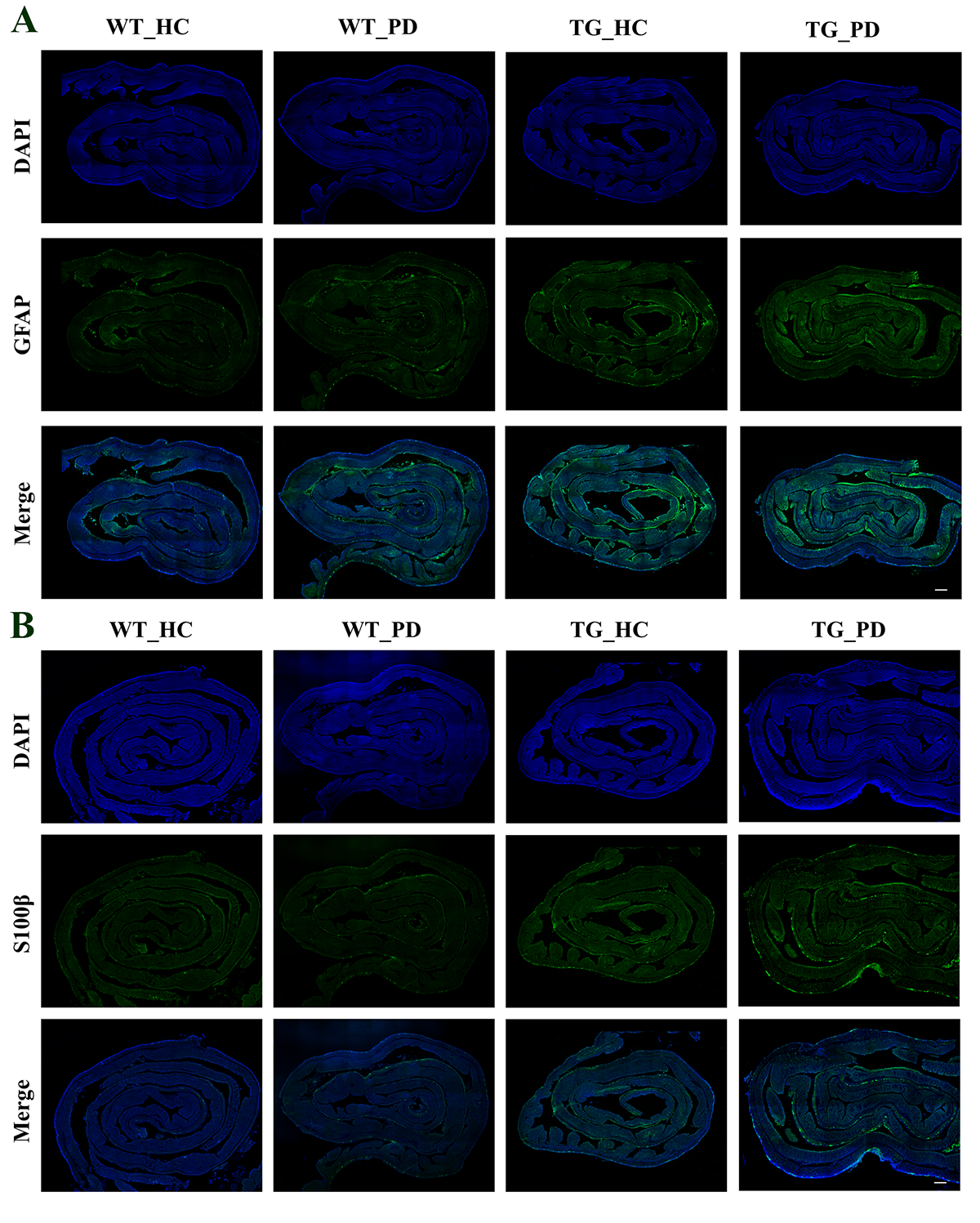


**Figure S4**

**FMT from PD patients induces inflammation in the colon of recipient mice. A** Representative images of GFAP immunofluorescence in the colon tissue. **B** Representative images of S100β immunofluorescence in the colon tissue, scale bar: 400 μm.


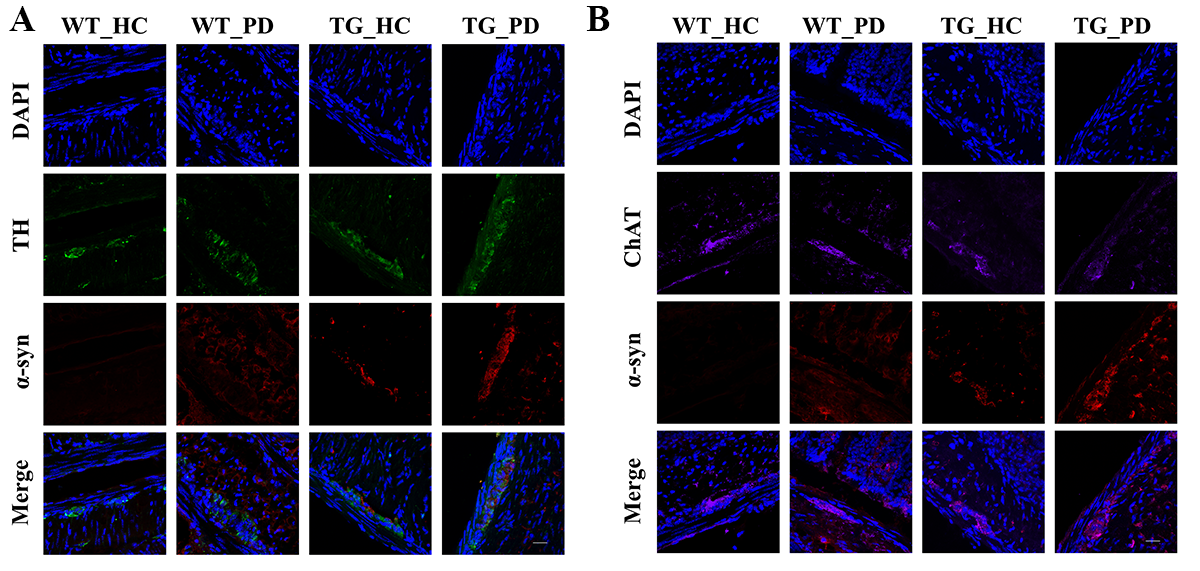


**Figure S5**

**α-syn detection in dopaminergic and cholinergic enteric neurons of the colon. A** Representative images of TH and α-syn immunofluorescence in the colon tissue. **B** Representative images of ChAT and α-syn immunofluorescence in the colon tissue, scale bar: 25 μm.


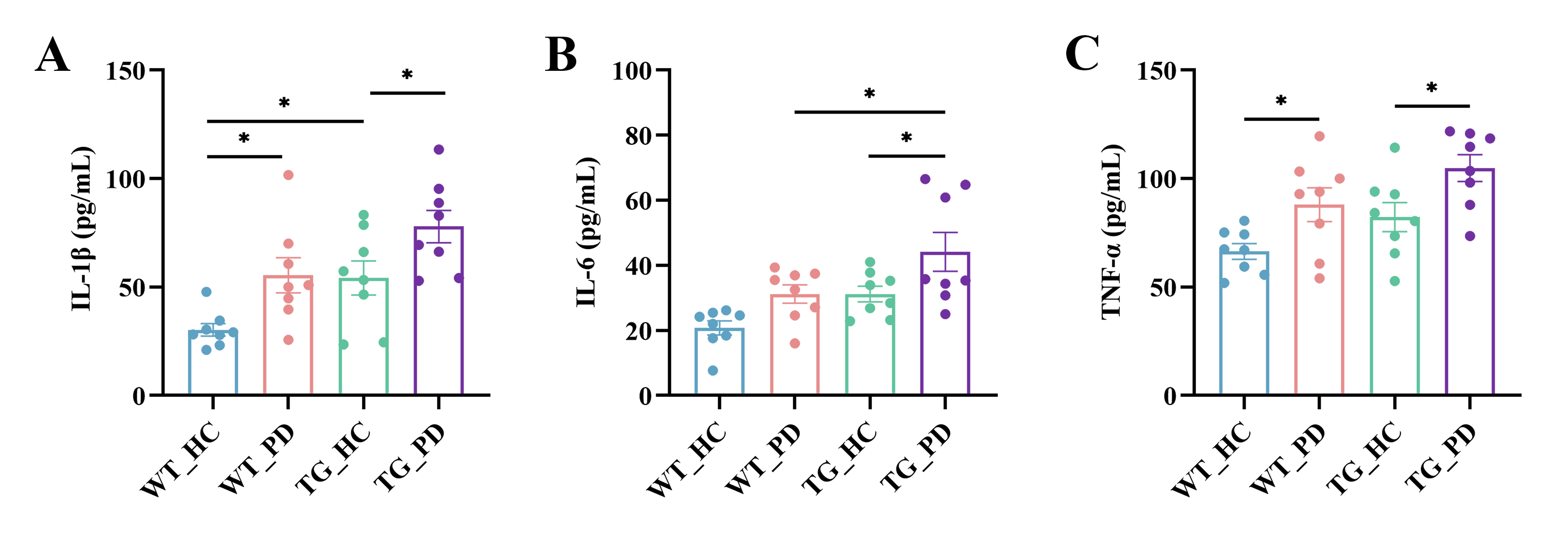


**Figure S6**

**FMT from PD patients increases circulating inflammation cytokines in recipient mice** **A-C** The expression levels of inflammatory factors, including IL-1β **(A)**, IL-6 **(B),** and TNF-α **(C)** in peripheral blood of recipient mice, n = 8 per group. Data are presented as mean ± SEM. *p* < 0.05 was set as the threshold for significance by two-way ANOVA followed by post hoc comparisons using Sidak’s test for multiple groups’ comparisons, **p* < 0.05.


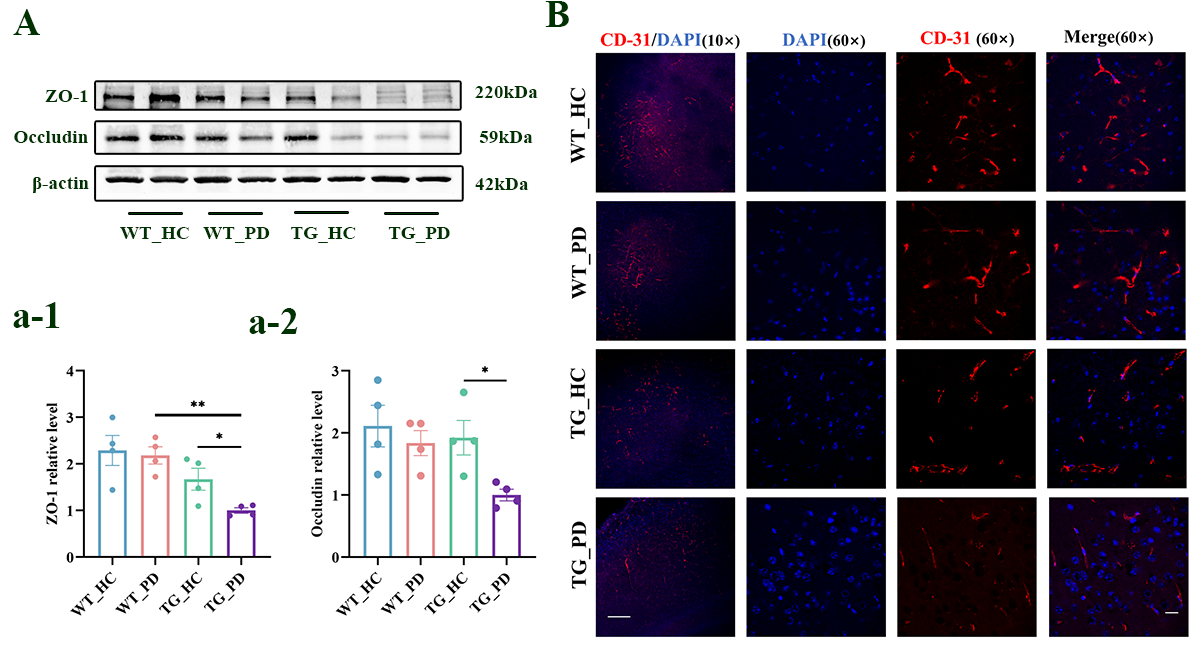


**Figure S7**

**FMT from PD patients induces BBB damage in the recipient mice. A** Representative western blot brands of ZO-1 and Occludin in the midbrain. **a** Relative quantitative results of ZO-1 **(a-1)** and Occludin **(a-2)** in the midbrain, n = 4 per group. **B** Representative immunofluorescence staining against CD-31 were performed in the cortex of recipient mice, the images in the leftmost column were acquired by 10× object lens, scale bar: 400 μm; the images in the right 3 columns were acquired by 60× object lens, scale bar: 25 μm. Data are presented as mean ± SEM. *p* < 0.05 was set as the threshold for significance by two-way ANOVA followed by post hoc comparisons using Sidak’s test for multiple groups’ comparisons, **p* < 0.05, ***p* < 0.01.


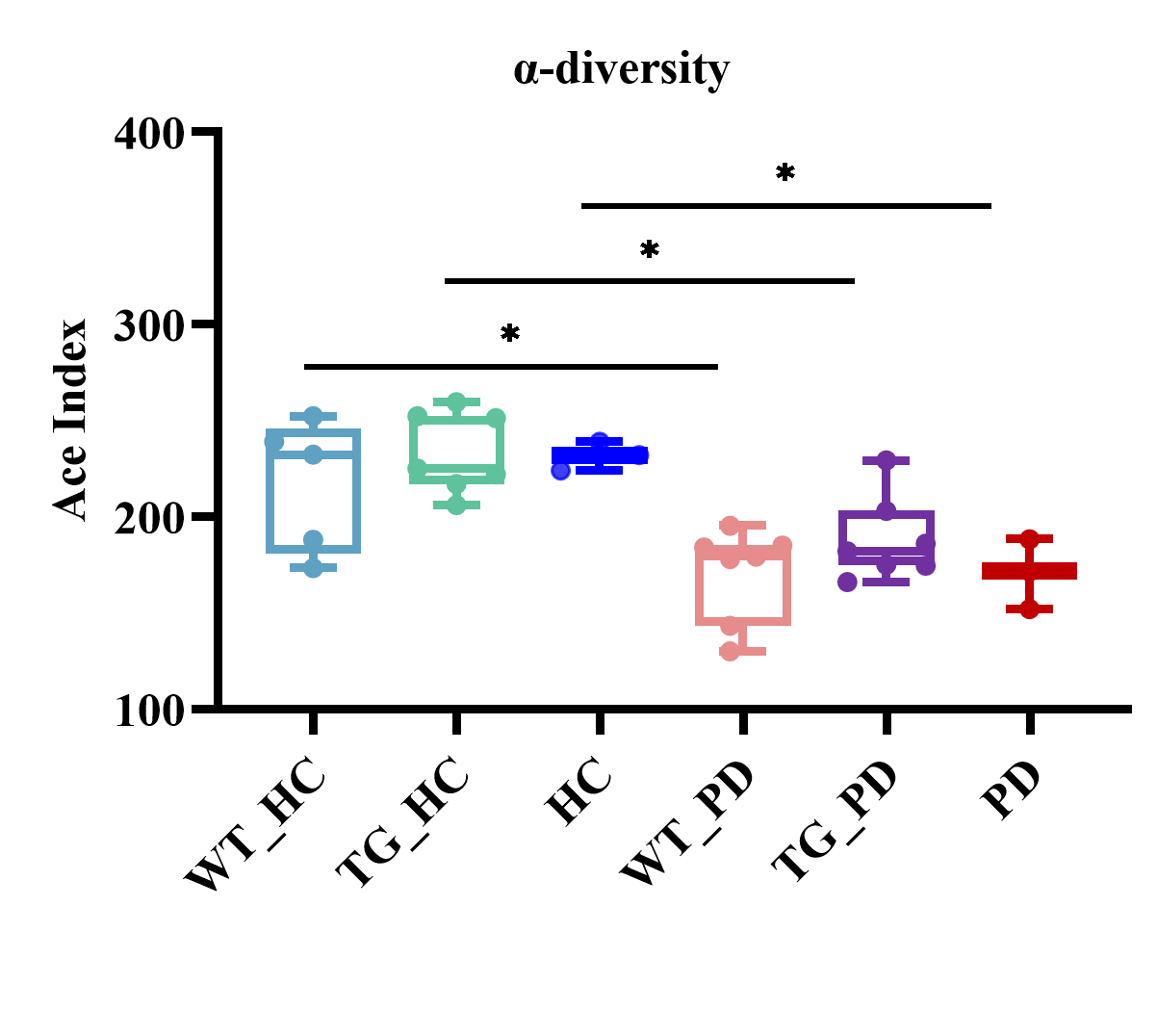


**Figure S8**

**The α-diversity in feces of donor and recipient mice.** The ace index in feces of donor and recipient mice. n = 3-7 per group. Data are presented as mean ± SEM. *p* < 0.05 was set as the threshold for significance by one-way ANOVA followed by post hoc comparisons using Tukey test for multiple groups’ comparisons, **p* < 0.05.


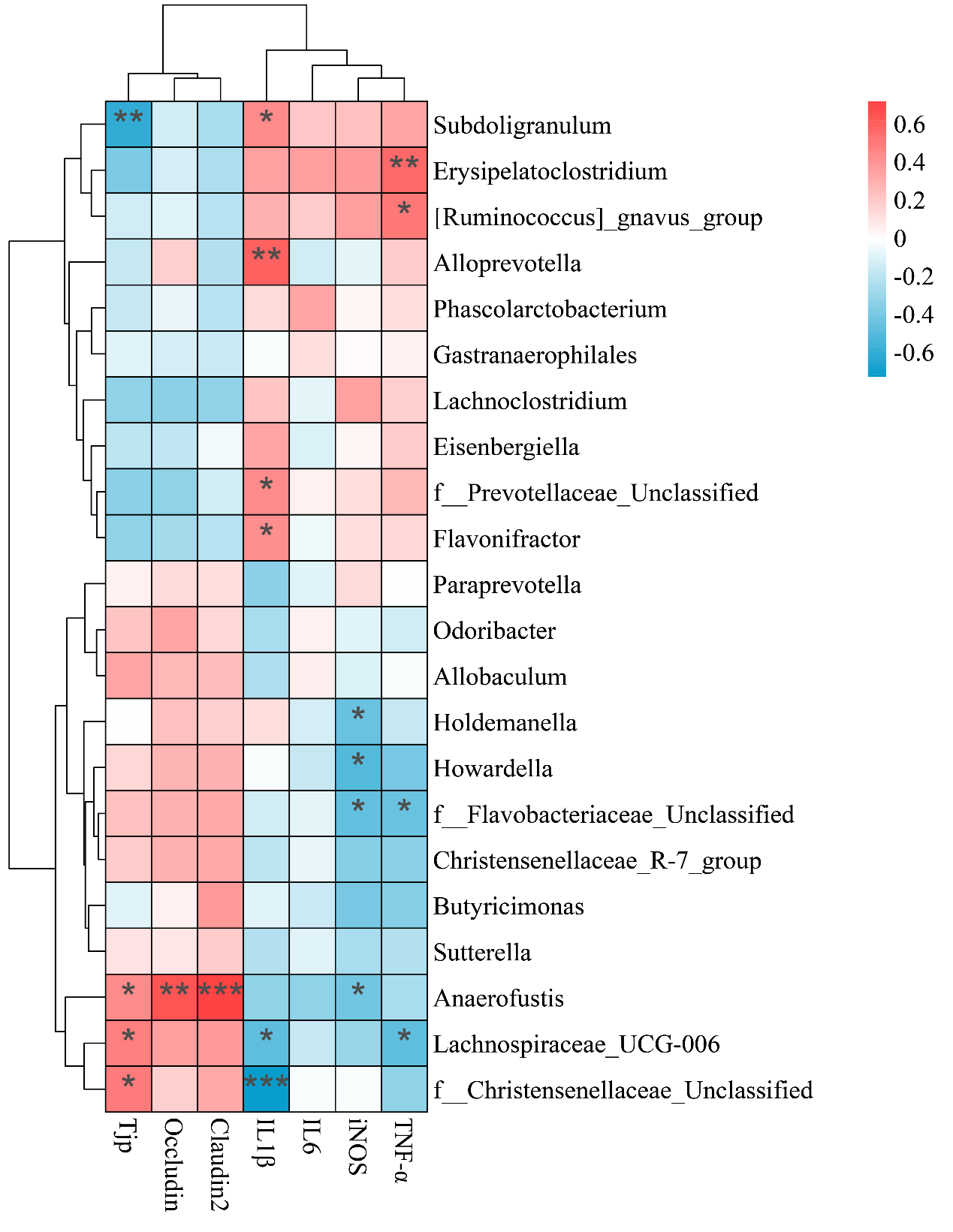


**Figure S9**

**Heatmap of the association between bacteria in genera and other experimental results.** The red color denotes a positive correlation, while blue color denotes a negative correlation. The intensity of the color is proportional to the strength of Spearman correlation. **p* < 0.05, ***p*< 0.01, ****p* < 0.001.


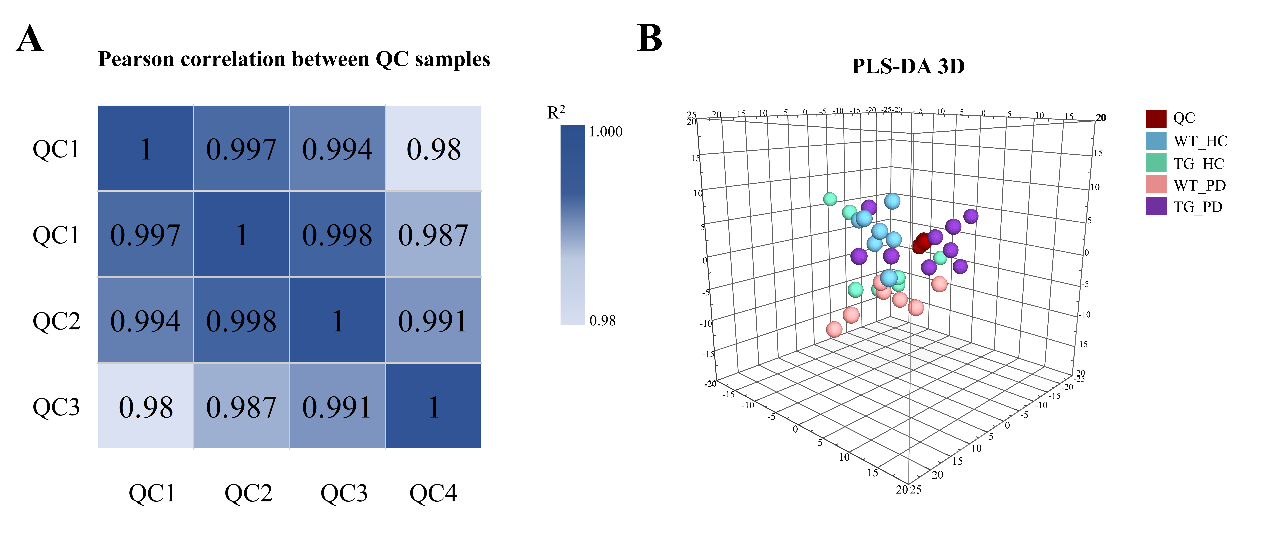


**Figure S10**

**Data quality checks for fecal metabolome.** **A.** The pearson-correlation of QC samples. **B.** Three-dimensional diagrams of PLS-DA of metabolic in fecal of different groups.


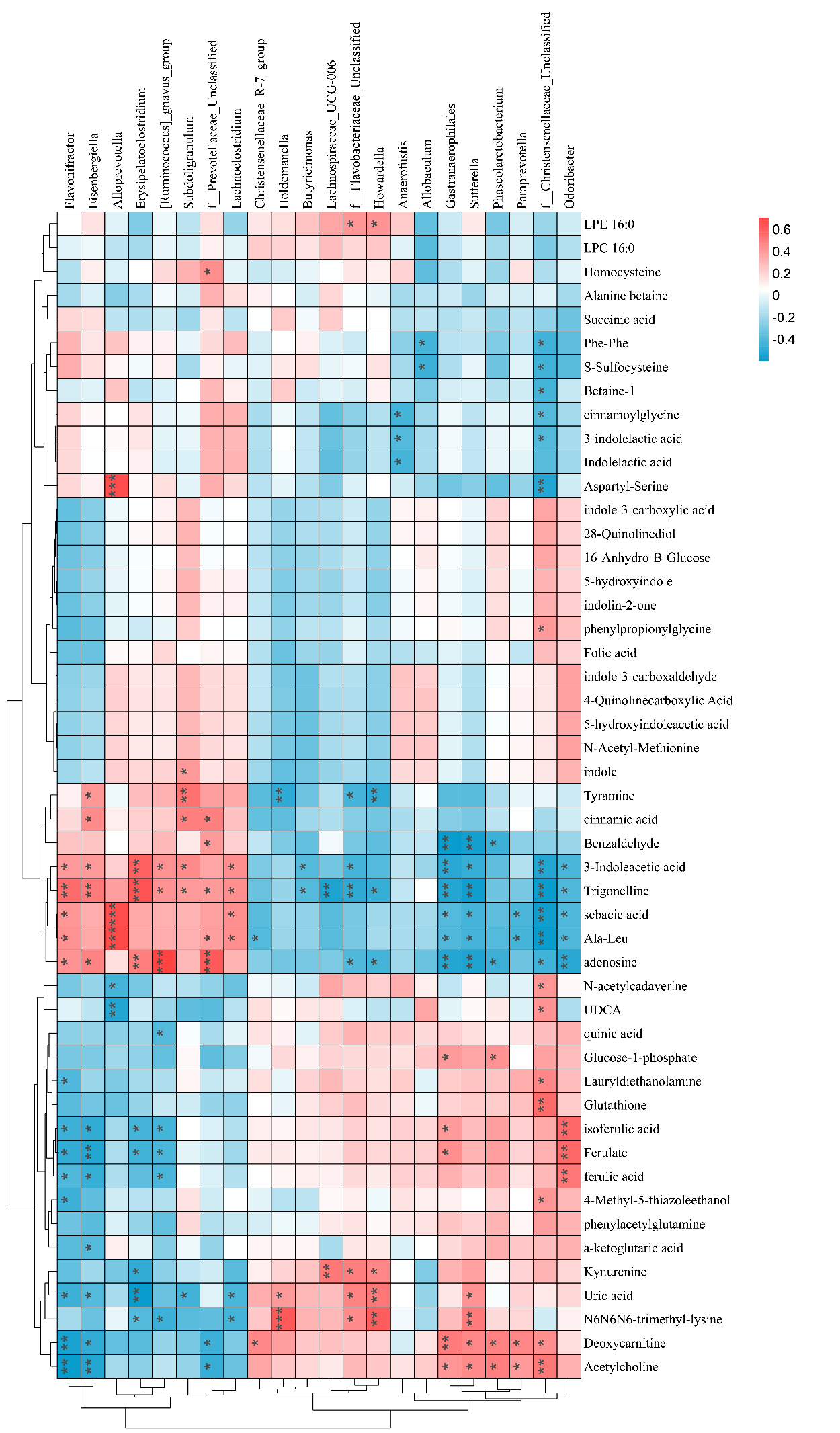


**Figure S11**

**Heatmap analysis of the Spearman correlation between fecal differential metabolites and dominating bacteria genera.** The red color denotes a positive correlation, while blue color denotes a negative correlation. The intensity of the color is proportional to the strength of Spearman correlation. **p* < 0.05, ***p*< 0.01, ****p* < 0.001.


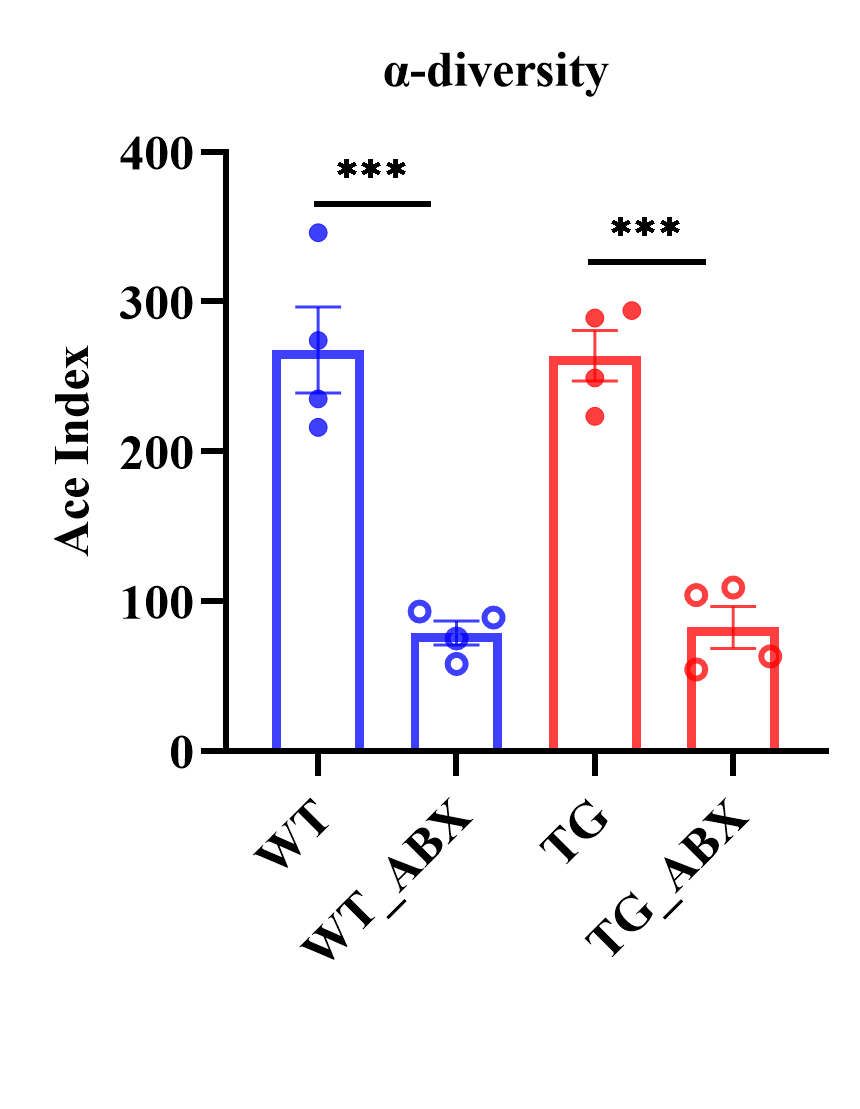


**Figure. S12**

**The α-diversity of mouse feces before and after antibiotic treatment.** The ace index in feces of mice before and after antibiotic treatment. n = 4 per group. Data are presented as mean ± SEM. *p* < 0.05 was set as the threshold for significance by two-way ANOVA followed by post hoc comparisons using Sidak’s test for multiple groups’ comparisons, **p* < 0.05, ***p*< 0.01, ****p* < 0.001.
